# A Review on Interface Engineering of MXenes for Perovskite Solar Cells

**DOI:** 10.1007/s40820-023-01083-9

**Published:** 2023-05-09

**Authors:** Srikanta Palei, G. Murali, Choong-Hee Kim, Insik In, Seul-Yi Lee, Soo-Jin Park

**Affiliations:** 1 Department of Chemistry, Inha University, 100 Inharo, Incheon, 22212 South Korea; 2 Department of Polymer Science and Engineering, Department of IT-Energy Convergence (BK21 Four), Chemical Industry Institute, Korea National University of Transportation, Chungju, 27469 South Korea

**Keywords:** MXenes, Perovskite solar cells, Additives, Interfacial layer, Electrodes

## Abstract

With an excellent power conversion efficiency of 25.7%, closer to the Shockley–Queisser limit, perovskite solar cells (PSCs) have become a strong candidate for a next-generation energy harvester. However, the lack of stability and reliability in PSCs remained challenging for commercialization. Strategies, such as interfacial and structural engineering, have a more critical influence on enhanced performance. MXenes, two-dimensional materials, have emerged as promising materials in solar cell applications due to their metallic electrical conductivity, high carrier mobility, excellent optical transparency, wide tunable work function, and superior mechanical properties. Owing to different choices of transition elements and surface-terminating functional groups, MXenes possess the feature of tuning the work function, which is an essential metric for band energy alignment between the absorber layer and the charge transport layers for charge carrier extraction and collection in PSCs. Furthermore, adopting MXenes to their respective components helps reduce the interfacial recombination resistance and provides smooth charge transfer paths, leading to enhanced conductivity and operational stability of PSCs. This review paper aims to provide an overview of the applications of MXenes as components, classified according to their roles as additives (into the perovskite absorber layer, charge transport layers, and electrodes) and themselves alone or as interfacial layers, and their significant importance in PSCs in terms of device performance and stability. Lastly, we discuss the present research status and future directions toward its use in PSCs.

## Highlights

- This review discusses the roles of MXenes in different positions/layers in perovskite solar cells.
- The issues in different layers/interfaces and their addressal with the incorporations of MXenes in perovskite solar cells are elaborately discussed.

## Graphical abstract

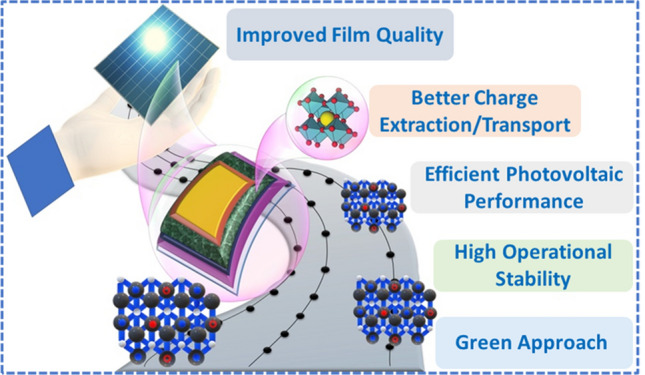

## Introduction

Solar technology converts solar energy directly into electrical energy through photovoltaic cells. Solar cells have attracted great attention from the energy community to meet the increasing demand for sustainable green, and clean energy sources. Apart from wafer-based Si solar cells that are widely available on the market, perovskite solar cells (PSCs) have achieved the highest power conversion efficiency (PCE) of 25.7% in 2021, which is inching towards the theoretical efficiency of about 31% [1–3]. Furthermore, a recent study shows halide PSCs can surpass the Shockley-Queisser (SQ) PCE limit of 33.7% (predicted for a single-junction solar cell) [4]. Most importantly, PSCs are in an advantageous position for ease of fabrication and their affordable cost coming from solution-based processing [5–7].

Perovskite materials, such as MAPbI_3,_ FAPbI_3_, and CsPbI_3_, have been spotlighted due to their excellent bandgap tunability, excellent light absorption *co*-efficient (> 10^5^ cm^−1^), good charge carrier mobility (2–40 cm^2^ V^−1^ s^−1^), and long carrier-diffusion length in the PSCs. Despite the stellar performance, the perovskite device remains a long-standing issue due to the structural and chemical instability originating from the degradation factors related to the intrinsic shortcomings of the perovskite materials as well as at their interfaces. Researchers have explored various pathways to improve stability and performance efficiency, for instance, the development of passivating materials, enlargement of the grain sizes or single crystals, solvent engineering, additive engineering, and interface engineering [6, 8–13].

Recently, MXenes, as emerging two-dimensional (2D) layered materials, have drawn interest in various applications, including photocatalysts, light emitting diodes, transparent electrodes, nanofiltration, water purification, electromagnetic interference shielding, antibacterial activity, supercapacitors, sensors, and more [14–22]. 2D nanomaterials such as graphene, g-C_3_N_4_, WS_2_, MoS_2_, and black phosphorous have been demonstrated as potential additives in the perovskite absorber layer in PSCs owing to their excellent electrical and optical properties [23–28]. MXenes have been used as all-round materials starting from additives, electron transport layers (ETL), hole transport layers (HTL), and interfacial layers to electrodes in PSCs due to their excellent electrical conductivity (2 × 10^4^ S cm^−1^), high charge carrier density (3.8 × 10^22^ cm^−3^), high mobility (1 cm^2^ V^−1^ s^−1^), high transparency, and tunable work function (WF) through controlled surface chemistry [29, 30]. With the introduction of Ti_3_C_2_T_*x*_ MXene into MAPbI_3_-based perovskite solar cells in 2018, there has been a tremendous surge in the use of MXenes in the photovoltaic community [31].

In this review, we aim to provide a comprehensive understanding of MXenes and their roles as additives or themselves alone in different components (classified according to photoactive absorber layer, ETL, HTL, and electrodes) and interfaces of the PSCs. In Sect. 1, we begin by introducing a brief overview of the recent research trend of PSCs. In Sect. 2, we have classified MXenes according to their roles as additives (into perovskite absorber layer, charge transport layers, and electrodes) and themselves alone in PSCs. In Sect. 3**,** we discuss MXenes as an interfacial layer on each layer of PSCs, followed by a discussion of MXenes’ roles in improving morphological features, electrical conductivity, mechanical stability, and WF tuning to impact electron transfer and extraction mechanism and thereby increasing the operational stability of PSCs. In Sect. 4, we suggest the future direction of an interfacial design/engineering that will realize the advent of an overarching framework for advanced PSC technology.

## MXenes in Perovskite Solar Cells

### Synthesis of MXenes

**Fig. 1:**
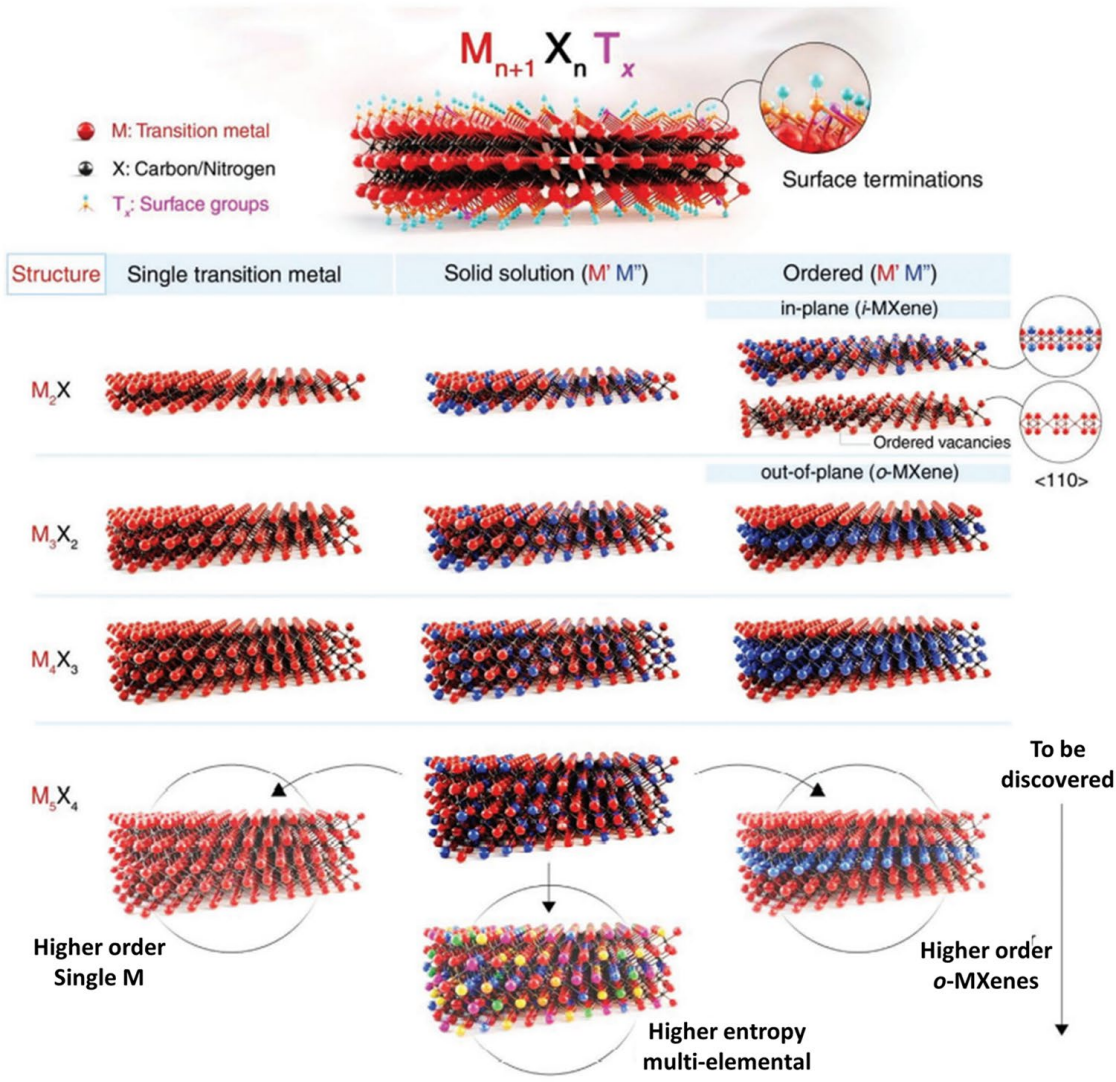
Schematic display for 2D MXene’s structures having a general formula of M_*n*+1_X_*n*_T_*x*_, (M = an early transition metal, X = carbon and/or nitrogen, and T_*x*_ = surface terminations of the outer metal layers, *n* = 1–4, examples: Ti_2_CT_*x*_, Ti_3_C_2_T_*x*_, Nb_4_C_3_T_*x*_, and (Mo, V)_5_C_4_T_*x*_) [37]. One or more transition metal atoms may occupy the M sites of MXenes to create ordered structures or solid solutions. The ordered double transition metal MXenes can be divided into three types: in-plane ordered structures (i-MXenes), in-plane vacancy structures, and out-of-plane ordered structures (o-MXenes), where either one layer of M′′ transition metal or two layers of M′′ transition metal are sandwiched between two layers of M′ transition metal. Other configurations might be feasible, such as the bottom row of the M_5_X_4_ structure with one, two, or three layers of M′′ sandwiched between layers of M′. Predicted structures like higher-order single M or o-MXenes and high-entropy MXenes that have not been empirically proven are represented by the faded images at the bottom of the schematic. Copyright © 2021 AAAS Publishing

MXenes are 2D transition metal carbides, nitrides, and carbonitrides with the general formula M_*n*+1_X_*n*_T_*x*_ (*n* = 1–4), where ‘M’ represents an early transition metal (Sc, Ti, Zr, Hf, V, Nb, Ta, Cr, Mo, etc.), ‘X’ represents carbon and/or nitrogen, and ‘T_*x*_’ stands for the surface terminations including –O, –OH, –F, etc*.* [32–34]. In 2011, Ti_3_C_2_T_*x*_ MXene was first reported by Naguib et al. [35]. MXenes with a distinct combination of M and X elements, number of elements at M or X sites (solid solutions at M or X sites, (M′,M′′)_*n*+1_X_*n*_T_x_, where M′ and M′′ are two different metals), number of atomic layers (*n*), ordering (in-plane or i- MXene or out-of-plane or o- MXene) of atoms, and compositions of surface functional groups (T_*x*_) were produced, as represented in Fig. 1 [36]. For certain combinations of transition metals, ordered MXenes are energetically more stable than their solid-solution counterparts. MXenes with monolayer or multilayer morphology are usually synthesized from the M_*n*+1_AX_*n*_ (MAX) phase precursors by eliminating monoatomic layers of the A element, which is from group 13 or 14 (*e.g*., Al, Ga, Si, or Ge). However, some of the MXenes are also synthesized from non-MAX phase precursors [37, 38]. The high reactivity of the A element and relatively weak strength of M–A bonds compared to M–X bonds allow the selective etching of A layers from the MAX phase, typically in aqueous solution using either direct hydrogen fluoride (HF) or in-situ HF (produced through LiF/HCl mixture) [33, 39, 40]. Over the years, apart from HF-based methods, such as using HF/other acids, HF/oxidants, NH_4_HF_2_, HCl/fluoride salt as etchants, several new synthesis methods include Lewis acidic molten salts assisted etching, alkali treatment under hydrothermal condition, treatment with organic bases, etching in organic solvents, salt-solution-based acoustic synthesis, electrochemical etching, etc*.* [41–56].

The type and composition of surface functional groups of MXene depend significantly on etching process, and they are mainly electronegative in nature, enabling to pull electron density away from the M atoms of MXenes (*i.e*., shifting the Fermi energy (${E}_{F}$) of MXenes to a lower energy). That is, the electronic structure and WF of MXenes can be easily tuned by controlling the surface functional groups' compositions [57, 58]. In addition to an ${E}_{F}$ shift, surface dipoles induced by surface functional groups play a significant role in tuning the WF of Mxenes [59, 60]. Computational calculations such as density functional theory (DFT) through altering the M and X elements and surface functional groups can also predict several other intriguing characteristics of MXenes, such as a wide range tunability of the WF between 1.6 and 8.0 eV, superconductivity, topological insulator behaviors, metal-to-semiconductor transition, and metal-to-insulator transition [59, 61–66]. Particularly, fine-tuning the WF brings a perfect energy offset between the perovskite active layer and the charge transport layer by adjusting the vacuum level and obtaining the optimal energy-level alignment at interfaces in PSCs applications. Optimizing energy offsets is necessary to achieve effective charge separation and/or collection. The various roles of MXenes as components themselves or additives into the components in PSCs, in terms of passivating defects, crystallinity, electrical conductivity, mechanical flexibility, and moisture resistance, are discussed in the following sections.

### PSCs

**Fig. 2:**
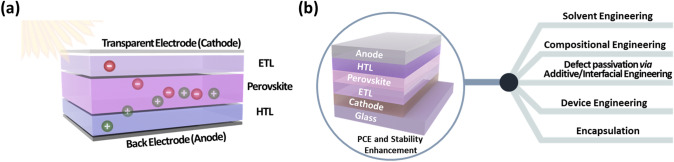
Schematics showing **a** stacking of different layers in a typical PSC and **b** the prominent ways for improving PCE and stability of PSCs

**Fig. 3:**
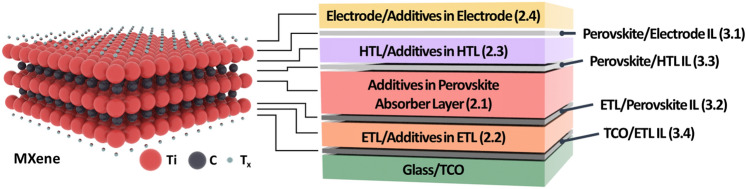
MXenes in PSCs**:** Various roles of MXenes as components or parts of the components in regular n-i-p PSCs. The device structure of Glass/TCO/ETL/Perovskite/HTL/Electrode with different ILs, positions of MXenes and numbers in the parenthesis indicate the Section numbers. IL stands for “interfacial layer,” and TCO stands for “transparent conducting oxide”

PSCs have been perceived as the next-generation photovoltaic candidates for commercial applications due to their high PCEs, easy and scalable solution fabrication processes, and low fabrication cost [67, 68]. For high-performance PSCs, the perovskite absorber layer is the most important. The characteristics such as crystallinity, surface coverage, compactness, uniformity, phase purity, internal trap defect density, grain size distribution, and grain boundaries of the perovskite absorber layers are the pivot players in determining the PCE performance of PSCs [69, 70]. A typical planar (or mesoporous) n-i-p or inverted p-i-n PSC consists of a photoactive absorber layer, an ETL, a HTL, and two (back and front) electrodes. The front electrode is based on a glass/flexible substrate facing simulated solar light. The solar light is incident on an absorber layer, and the electrons excite from the valence band to the conduction band, forming electron–hole pairs [8, 71]. These photogenerated charge carriers are extracted by the respective ETL and HTL and then moved to the electrodes (Fig. 2a). These distinctive features determine the stability and current–voltage hysteresis. Several key factors such as bulk, surface defects and reduced charge extraction/transportation, moisture, humidity, heat, and electric field can impede the solar cell device performance and longevity [72]. These impediments can be controlled via solvent engineering, additive engineering, compositional engineering, interface engineering, device engineering, and encapsulation (Fig. 2b**)** [8, 73–76]. Furthermore, until now, the highest efficiency devices are Pb-based PSCs. Due to the Pb toxicity, several alternatives such as lead-free PSCs, double-PSCs, and 2D Ruddlesden-Popper (RP) and Dion-Jacobson (DJ) PSCs have been examined as promising candidates in the perovskite photovoltaic arena [77–80]. We have excluded an insightful discussion on the topics mentioned above and suggest you refer to this paper [72]. In this review, we discuss various roles of MXenes as components or parts in PSCs, as depicted in the schematic (Fig. 3). Thus, we categorized the functions of MXenes depending on the positions and the roles of MXenes as additives in the perovskite absorber layer/ETL/HTL/electrodes or as ETL/HTL/electrodes along with as interfacial layers. The discussion about their respective pros and cons is presented in the following sections.

### MXenes as Additives in Perovskite Absorber Layers

**Fig. 4:**
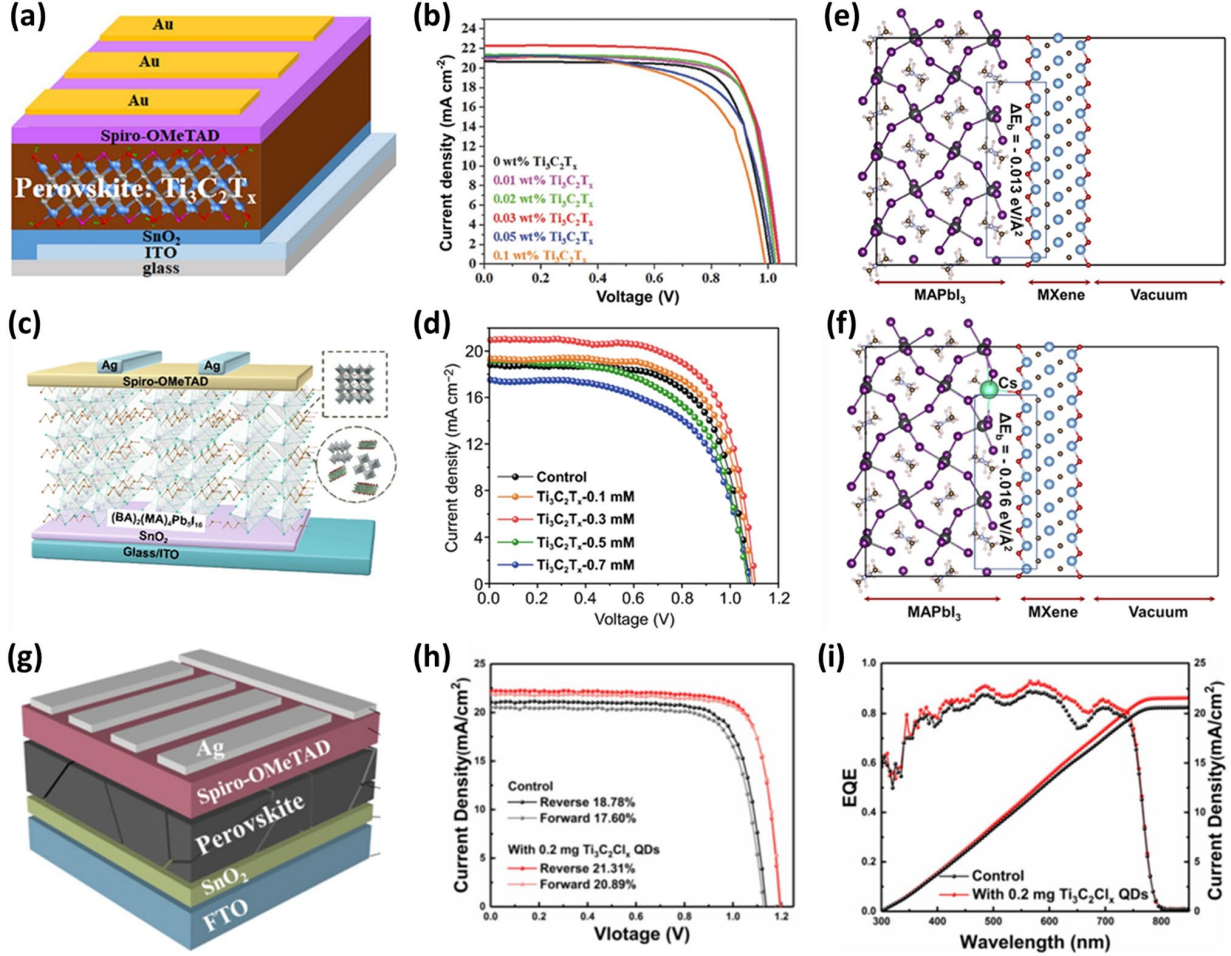
**a** Schematic of MAPbI_3_ planar PSCs with MXenes additives in the absorber layer. **b** *J–V* characteristics curves of PSCs with different amounts of Ti_3_C_2_T_*x*_ [31]. Copyright © 2018 WILEY–VCH Verlag GmbH & co. KGaA, Weinheim. **c** Schematic diagram of PSCs with the structure of Glass/ITO/SnO_2_/2D perovskite/Spiro-OMeTAD/Ag.** d** *J–V* curves of PSCs with different amounts of Ti_3_C_2_T_*x*_ doping [96]. Copyright © 2018 Springer. **e** The atomic structures showing the interface between the MAPbI_3_ (100) surface and the Ti_3_C_2_O_2_ MXene without Cs^+^ and **f** with Cs^+^ [97]. Copyright © 2021 Cell Press. **g** Schematic illustration of the device structure. **h** Reverse and forward scans for the pristine and 0.2 mg mL^−1^ Ti_3_C_2_Cl_*x*_ QDs-treated PSC devices. **i** EQE of the devices (the pristine and 0.2 mg mL^−1^ Ti_3_C_2_Cl_*x*_ QDs-treated) [98]. Copyright © 2022 Elsevier B.V

First up, the PCE performance of a PSC depends on the light-absorbing perovskite film layer that generates the charger carriers (electrons and holes). The charge carriers are separated and collected at the respective electrodes [81]. The quality of the perovskite film is determined by crystal sizes and grain boundaries. Large crystal sizes with fewer grain boundaries are essential for maximum charge transfer and high-performance [82, 83]. In 2018, Guo et al*.* introduced the Ti_3_C_2_T_*x*_ MXene into a CH_3_NH_3_Pbl_3_ (MAPbI_3_) perovskite absorber layer [31]. This is the first report on an MXene’s application in a PSC. Ti_3_C_2_T_*x*_ MXene slows the crystallization rate and enlarges the size of MAPbI_3_ crystals with no pin holes. The O–H⋅⋅⋅I^−^ van der Waals interaction between the methylammonium iodide (MAI) and the additive restricts uniform nucleation on the tin oxide (SnO_2_) layer and rather generates lesser nuclei, implying slow crystal growth or retardation of the nucleation process [84]. After annealing, the small crystals grow into large crystals. Perovskite films with Ti_3_C_2_T_*x*_ MXene additives show an improvement in light absorption owing to enhanced scattering of incident light from the large crystals [85, 86]. PSC with a device structure of indium tin oxide (ITO)/SnO_2_/MAPbI_3_:(0.3 wt%) Ti_3_C_2_T_*x*_/2,2′,7,7′-tetrakis(N,N-di-p-methoxyphenyl-amine)9,9′-spirobifluorene (Spiro-OMeTAD)/Au achieved a PCE of 17.4%, which is 12% higher than the PCE (15.6%) of the control device. The device with the optimal amount of additives also showed an increased open-circuit voltage (*V*_*oc*_), current density (*J*_*sc*_), and fill factor (FF). The increase in *V*_*oc*_ is ascribed to the passivation of the perovskites by Ti_3_C_2_T_*x*_, spurring the hole extraction and reduced recombination at the perovskite/HTL interface [87]. A schematic of a PSC with Ti_3_C_2_T_*x*_ additive and *J–V* curves with 0, 0.01, 0.02, 0.03, 0.5, and 1 wt% of Ti_3_C_2_T_*x*_ MXene additives are shown in Fig. 4a–b. The optimal amount of additive is 0.03 wt%. On further increase in wt%, the additives form aggregates on the surface of the perovskite films, resulting in charge trapping centers. Hence, the device performance degrades with excessive additives. The charge transfer resistance of the perovskite film with additive also reduced from 7000 to 1800 Ω, indicating fast charge transfer, and hence the *J*_*sc*_ was increased [31]. All-inorganic CsPbI_3_ perovskite has a near-ideal bandgap and thermal stability and is a suitable candidate for the development of perovskite/Si tandem solar cells. But when exposed to moisture, CsPbI_3_ experiences a quick phase change. Especially, the preferred p-i-n structure for perovskite/Si tandems shows a large performance difference compared to other perovskite compounds. Heo et al. reported a surface-engineered CsPbI_3_ layer with oxidized Ti_3_C_2_T_*x*_ MXene (OMXene) nanoplates to form effective and stable p-i-n-structured CsPbI_3_ perovskite solar cells [88]. In addition to improving charge separation at the perovskite-electron transporting layer interface through an improved electric field, OMXene acts as a physical barrier against moisture.

As a result, the p-i-n devices based on CsPbI_3_/OMXene dispersion with 10 wt% concentration attained the PCEs of 19.7% for 0.096 cm^2^ cells and 14.6% for 25 cm^2^ in minimodules. The pristine cell has a PCE of 18.1% with a *V*_*oc*_, *J*_*sc*_, and FF of 1.18 V (1.21 V), 19.05 (19.85) mA cm^−2^, and 80.3% (81.6%), respectively. Furthermore, the encapsulated minimodule showed good stability, maintaining 85% of the initial efficiency over 1000 h while being exposed to 85 °C at a relative humidity (RH) of 85% and 1-sun light soaking. Zhou et al*.* reported an inorganic Cl-terminated Ti_3_C_2_ (Ti_3_C_2_Cl_*x*_) MXene to passivate the surface defect states and grain boundaries of CsPbBr_3_ film. The addition of Ti_3_C_2_Cl_*x*_ MXene enlarges the crystal sizes. The solvent volatilization produced the Ti_3_C_2_Cl_*x*_-tailored PbBr_2_ film with a high porosity [89]. CsBr can be diffused into these pores to grow the perovskite grain with sizes compared to pre-expanded volume and thus result in a high-quality CsPbBr_3_ perovskite film with large and compact grains. The residual stress in the soft perovskite lattice distorts the surface lattices, leading to low device performance. Introducing Ti_3_C_2_Cl_*x*_ MXene removes the residual stress owing to a strong Pb–Cl binding energy (301 kJ mol^−1^), paving the way for a high device efficiency [90]. In further work, Saranin et al. introduced MXene doping into the perovskite absorber layer in inverted perovskite solar cells [91]. MXene doping in the absorber layer significantly decreases charge recombination caused by deep trap states. More charges are collected at the perovskite/phenyl-C_61_-butyric acid methyl ester (PCBM) interface compared to reference devices. The MXene-based tailored cells outperformed the pristine devices and exhibited PCEs of 18.6% and steady power output.

2D RP PSCs have received significant interest owing to their exceptional stability against moisture. The bulky cations in 2D RP perovskites restrict the internal movement of ions and allow the passage of organic ions, thus resulting in the hydrophobic nature of the absorber layer [92, 93]. However, low crystallinity, disordered orientation, and inferior charge transport constrain the PCE of 2D PSCs [94, 95]. Jin et al. explored Ti_3_C_2_T_*x*_ MXene nanosheets as additives in 2D RP (BA)_2_(MA)_4_Pb_5_I_16_ perovskite absorber layer to fabricate the perovskite films with excellent electrical conductivity and mobility [96]. A homogeneous and highly crystalline perovskite film with Ti_3_C_2_T_*x*_ MXene nano-dopants was formed. After adding an optimal concentration of 0.3 mM Ti_3_C_2_T_*x*_ nanosheets, the PCE of PSC with a configuration of ITO/SnO_2_/(BA)_2_(MA)_4_Pb_5_I_16_-Ti_3_C_2_ MXene/Spiro-OMeTAD/Ag increased from 13.7% (without additive) to 15.7%. The Ti_3_C_2_T_*x*_ nanosheets doped-PSC also exhibited an increased *J*_*sc*_ of 20.87 mA cm^−2^, *V*_*oc*_ of 1.11 V, and FF of 67.8%. The schematic of the PSC, 2D RP layered structure, and *J–V* curves of the PSC with different concentrations are shown in Fig. 4c–d. The enhanced crystallinity, orientation, and passivated trap states led to an accelerated charge transfer process in the vertical direction and are responsible for this improved performance. The unencapsulated PSCs with Ti_3_C_2_T_*x*_ nanosheets exhibited excellent stability in ambient settings with an RH of 55% [96].

In 2021, Bati et al. produced Ti_3_C_2_T_*x*_ MXene nanosheets doped with cesium (Cs) and added them to a lead iodide (PbI_2_) precursor solution for PSCs via a two-step deposition approach [97]. The theoretical and practical study demonstrated that Cs is crucial for enhancing PCE. The perovskite crystallization with doped Ti_3_C_2_T_*x*_ MXene results in larger crystal grains, long lifetime charge carriers, and reduced charge recombination. The PSCs combined with Cs-doped Ti_3_C_2_T_*x*_ Mxene (ITO/SnO_2_/FA_1-*x*_MA_x_PbI_3-*x*_Br_*x*_:Cs-Ti_3_C_2_T_*x*_/Spiro-OMeTAD/Au) achieved a high PCE of 21.6% (19.0% for the pristine device) with increased *V*_*oc*_, *J*_*sc*_, and FF. The increased *V*_*oc*_ is ascribed to the filling of Cs^+^ ions into the surface MA^+^ vacancy. Moreover, with the introduction of Cs^+^_,_ the interaction between MXene and perovskite film increases with a binding energy of −0.016 eV Å^−2^. In contrast, without Cs^+^, the binding energy between the perovskite film and MXene is −0.013 eV Å^−2^ (Fig. 4e–f). Additionally, the unencapsulated Cs- Ti_3_C_2_T_*x*_ MXene-doped PSC device exhibited stability by retaining 65% of its initial PCE after 2000 h [97].

High photovoltaic performance and large-scale commercialization of planar PSCs depend on the perovskite layer having a high crystallinity and long-term stability against high humidity. In 2021, Liu et al. used one-step deposition to incorporate Ti_3_C_2_Cl_*x*_ quantum dots (QDs) as additives in the perovskite precursor solution [98]. During the film crystallization process, the strong interaction between the Cl terminations of Ti_3_C_2_Cl_*x*_ QDs and Pb^2+^ ions slowed down the crystallization rate and induced the preferred grain orientation. This resulted in a high-quality perovskite film with high crystallinity, fewer trap-states, and less residual tensile strain. Ti_3_C_2_Cl_*x*_ QDs also quickened charge extraction and benefitted band alignment between the SnO_2_ ETL and the perovskite layer due to their top–bottom rising gradient distribution. As a result, the PSC with 0.2 mg mL^−1^ Ti_3_C_2_Cl_*x*_ QDs and a device structure of fluorine-doped tin oxide (FTO)/SnO_2_/Perovskite + Ti_3_C_2_Cl_*x*_ QDs/Spiro-OMeTAD/Ag) achieved an improved efficiency of 21.3% (18.8%) and *V*_*oc*_ of 1.19 V with minimal hysteresis (Fig. 4g–h). The Cl-terminated Ti_3_C_2_ QDs can also prevent potential deprotonation of protonated organic amine in the perovskite, improving the overall stability. The Ti_3_C_2_Cl_*x*_ QDs (0.2 mg mL^−1^) device shows a higher integrated *J*_*sc*_ of 21.60 mA cm^−2^ than the pristine device (20.64 mA cm^−2^). The calculated *J*_*sc*_ is less than the observed *J*_*sc*_ by less than 5% discrepancy (Fig. 4i). Additionally, the unencapsulated device demonstrates exceptional long-term humidity stability by retaining over 84% of its initial PCE after aging for 1000 h at 40% RH in the dark at room temperature [98].

**Fig. 5:**
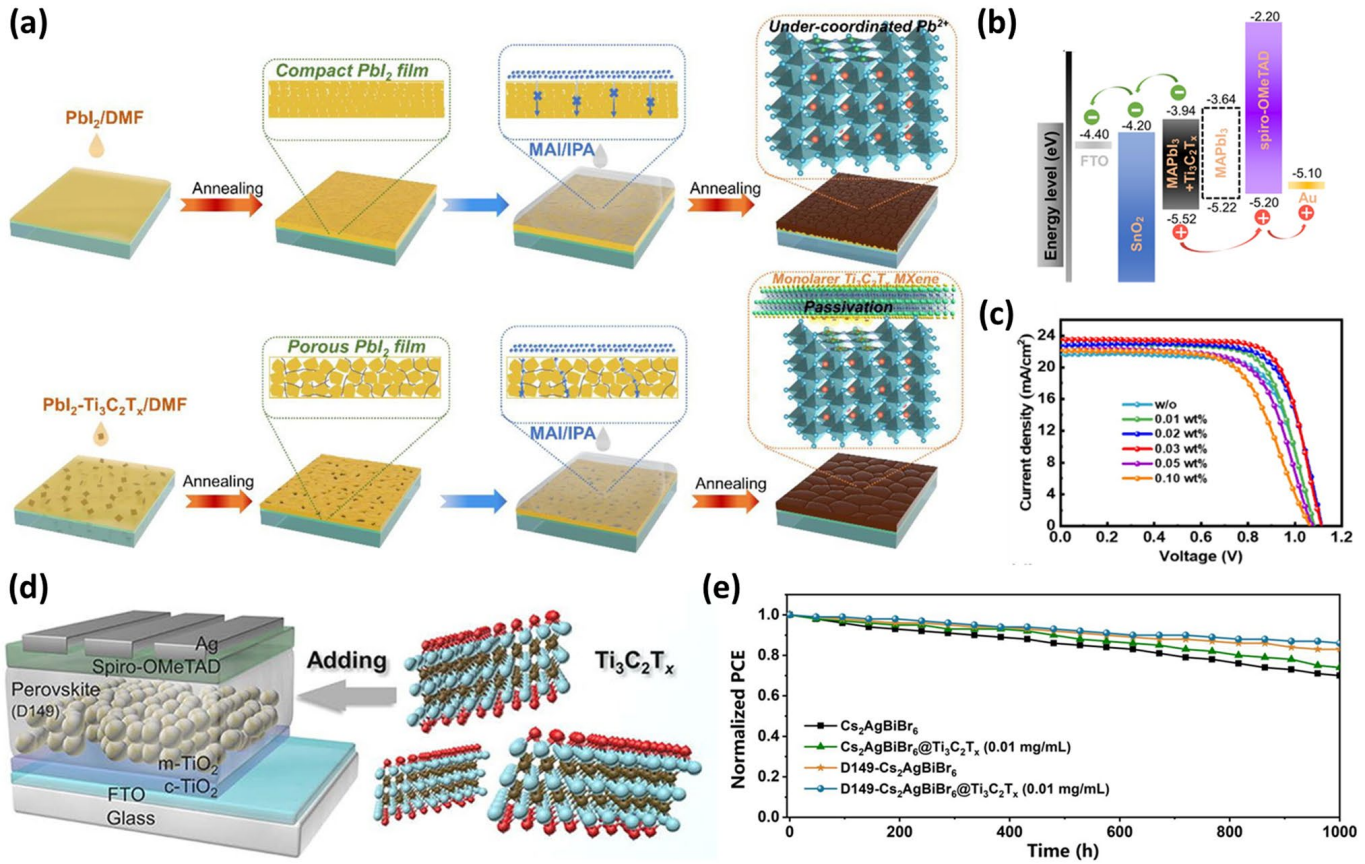
**a** Mechanism diagram illustrating the preparation of high-quality two-step-processed perovskite films assisted by Ti_3_C_2_T_*x*_ additive. **b** Device configuration of the planar PSCs and its energy-level diagram with and without Ti_3_C_2_T_*x*_ doping. **c** *J–V* curves of the champion PSCs with different contents of Ti_3_C_2_T_*x*_ [106]. Copyright © 2021 Elsevier B.V. **d** Schematic of the PSC with Ti_3_C_2_T_*x*_ MXene additive in the perovskite absorber layer. **e** The stability of the PSC devices with and without D149 sensitized TiO_2_ and/or Ti_3_C_2_T_*x*_ doping in ambient air at RH ~ 20% without encapsulation at 25 °C [107]. Copyright © 2022 Elsevier B.V

The solution method is the most common route to fabricate the perovskite films. The solution method is categorized into one-step and two-step sequential processes [99–101]. In the simple and facile one-step solution method, it is difficult to achieve uniform and compact perovskite films owing to the anisotropic growth of perovskites [102]. In the two-step process, the PbI_2_ layer is formed first, and then the MAI solution is dropped to react, and thus high-quality perovskite films without cracks and pinholes are formed. Crack and pinhole-free perovskite films are pertinent for highly efficient PSCs [103]. However, low reactivity leads to incomplete conversion of PbI_2_, and hence compact films are not formed owing to the presence of residual PbI_2_. As a result, the device performances are degraded [104, 105]. In 2021, to increase the reactivity of PbI_2_ by generating porous channels, Zhao et al. added 2D monolayer Ti_3_C_2_T_*x*_ (T_*x*_ = –O, –OH, and –F) MXene nanosheets into the PbI_2_ layer [106]. This modification enhanced the subsequent PbI_2_ reaction with MAI to transform into MAPbI_3_ completely. As a result, the quantity of remaining PbI_2_ in the perovskite film was reduced, and the size of the perovskite grain increased (Fig. 5a). Additionally, Ti_3_C_2_T_*x*_ MXene controls the perovskite WF, resulting in a better energy-level alignment that makes carrier extraction and injection easier (Fig. 5b). The functional groups on the surface of Ti_3_C_2_T_*x*_ were found to interact with the poorly coordinated Pb^2+^ in perovskites to passivate defects, having a significant impact on reducing hysteresis and inhibiting nonradiative recombination. Finally, by adding 0.03 wt% of the Ti_3_C_2_T_*x*_ additive, the PSC with a device configuration of FTO/SnO_2_/MAPbI_3_:Ti_3_C_2_T_*x*_/Spiro-OMeTAD/Au achieved a PCE of 19.3%, demonstrating an improvement of about 18% over the control device (Fig. 5c) [106].

A promising contender for inorganic double perovskite solar cells without lead is Cs_2_AgBiBr_6_ PSC. The reported PCE of Cs_2_AgBiBr_6_ is approximately 3%, which limits its photovoltaic capability. Yang et al. used D149 indoline dye to make the TiO_2_ ETL more sensitive and Ti_3_C_2_T_*x*_ MXene nanosheets to Cs_2_AgBiBr_6_ to improve the crystallization [107]. Cs_2_AgBiBr_6_ and D149 indoline dye both have the potential to influence the photocurrent. Furthermore, the DFT was used to calculate the interface properties and electron structure. Fermi level pinning is effectively reduced while the perovskite maintains its semiconductor properties, thanks to weak van der Waals forces at the interfaces of Cs_2_AgBiBr_6_ and Ti_3_C_2_T_*x*_. The high WF of Ti_3_C_2_T_*x*_ modifies the Fermi level of the valence band of Cs_2_AgBiBr_6_, which results in increased carrier mobility. The device showed enhanced long-term stability and PCEs of 4.5% under 1-sun illumination and 7.2% under 200 lx indoor light illumination. Furthermore, the unencapsulated devices based on Cs_2_AgBiBr_6_, Cs_2_AgBiBr_6_@ Ti_3_C_2_T_*x*_ (0.01 mg mL^−1^), D149-Cs_2_AgBiBr_6_ and D149-Cs_2_AgBiBr_6_@Ti_3_C_2_T_*x*_ (0.01 mg mL^−1^) exhibited long-term stability by retaining 70%, 74%, 83%, and 86% of their initial PCEs after 1000 h of storage at 25 °C in air at about 20% RH (Fig. 5d–e) [107]. Bykkam et al. used a 2D MXene as an additive in MAPbI_3_ perovskite at concentrations ranging from 0 to 20 vol% with a 5 vol% increment [108]. With an increase in the vol.% of the 2D MXene, the perovskite peak at 2θ = 14.2° shifts slightly towards the lower angle. The continuous change in peak position reflects the homogeneous distribution of strain during perovskite crystal formation and stresses induced by the 2D MXene additive. However, the perovskite film with 5 vol% additive has fewer defects and improves the photoresponse of the PSC. On further increase in additive concentration, stacking of 2D MXene occurs in the perovskite active layer, hindering the light propagation across the perovskite film and reducing the photogeneration of charge carriers. The best PSC device with 5 vol% 2D MXene additive attained the highest PCE of 13.6%, *V*_*oc*_ of ~ 0.81 V, *J*_*sc*_ ~ 27.6 mA cm^−2^, and FF of ~ 61.1%. This is higher than the PCE of 11.4% in the PSC without 2D MXene [108].

Din et al. explored the synergistic effect of high-quality NiO_*x*_ HTLs deposited by ion beam sputtering on ITO substrates and the Ti_3_C_2_T_*x*_ MXene doping of MAPI perovskite layers to increase the PCE of p-i-n PSCs [109]. The 18 nm-thick NiO_*x*_ films are pinhole-free and have large-scale uniform surface morphology. For non-stoichiometric NiO_*x*_, the grazing-incidence X-ray diffraction revealed a 0.75% enlargement of the face-centered cubic lattice. Atomic force microscopy studies revealed that doping increased the size of MAPI polycrystalline grains from 430 ± 80 to 620 ± 190 nm. The best PSC with 0.15 wt% MXene doping with a device structure of ITO/NiO_x_/MXene-doped MAPI/PC_61_BM/BCP/Ag showed a PCE of 16% (14% for the undoped PSC), which is a 14.3% improvement. However, the band gap of the MXene-doped MAPI layer was found to have a one order of magnitude higher density of defect states (∼10^19^ cm^−3^ eV^−1^ for the MXene-doped MAPI and ∼10^18^ cm^−3^ eV^−1^ for the undoped-MAPI), which lowers the favorable effect of the total area of bigger MAPI grain boundaries, reducing the *J*_*sc*_ of the MXene-doped devices. The WF drops from −5.26 to −5.32 eV with MXene doping, which raises the *V*_*oc*_ and FF, and thus is credited with improving the PCEs of PSCs. Such different results are attributed to large and partly delaminated multilayer MXene sheets and an increased density of states in the bandgap [109].

To improve the PCE of PSCs using a one-step coating method, Li et al. added 2D Ti_3_C_2_T_*x*_ and V_2_CT_*x*_ MXene to a PbI_2_ precursor solution to fabricate perovskite films [110]. The addition of Ti_3_C_2_T_*x*_ and V_2_CT_*x*_ additives boosted the hydrophobicity of the perovskite film, showing water contact angles of 85.4° and 69.8°, respectively, as compared to 52.9° for the pristine film. Furthermore, the additives also enhanced the shape and grain size of the perovskite films. The PCEs of PSCs with Ti_3_C_2_T_*x*_ and V_2_CT_*x*_ additives attained 17.6% and 17.2%, respectively, compared to the device without additives (15.0%). Moreover, a distinct morphology with uniform grain size and organized layered crystal particles can be observed for perovskite films employing the V_2_CT_*x*_ additive, indicating that the V_2_CT_*x*_ additive controls the formation of perovskite crystal films. Due to the improved crystallinity and perovskite film quality, the V_2_CT_*x*_ additive integrated devices can preserve 68.3% of the initial PCE after 15 days, which is greater than the devices without additives by 9.7% and with Ti_3_C_2_T_*x*_ as additive by 47.2%. Thus, V_2_CT_*x*_ in photovoltaics offers a practical way to enhance the performance of PSCs [110].

### MXenes as ETLs or Additives in ETL

#### MXenes as ETLs

**Fig. 6:**
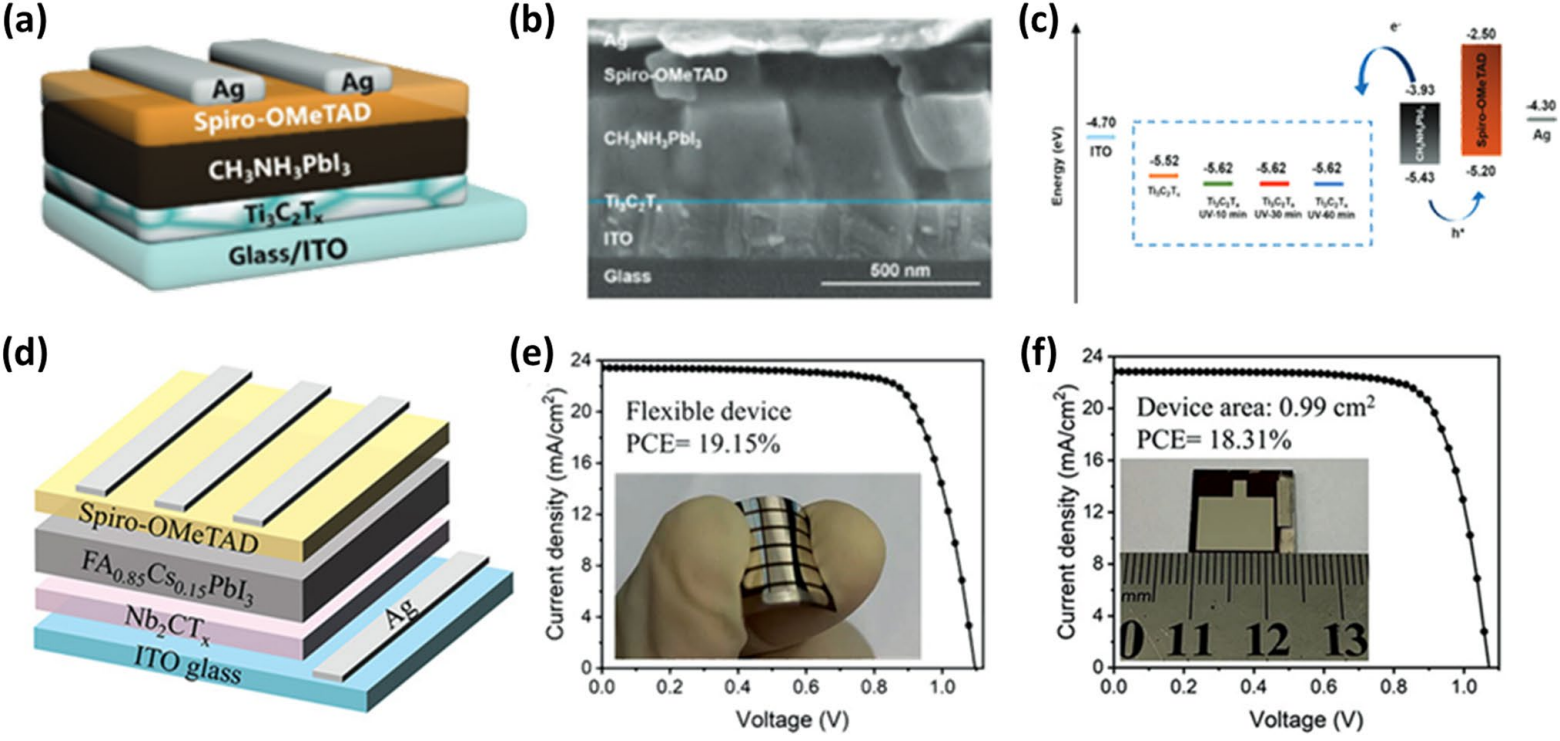
**a** Device architecture of ITO/ETL/MAPbI_3_/Spiro-OMeTAD/Ag based on Ti_3_C_2_T_*x*_ with/without UV-ozone treatment as ETL. **b** Cross-sectional SEM image of the PSC device. **c** Schematic energy-level diagram of each layer [111]. Copyright © 2019 WILEY–VCH Verlag GmbH & Co. KGaA, Weinheim.** d** Schematic device structure of ITO/Nb_2_CT_*x*_/FA_0.85_Cs_0.15_PbI_3_/Spiro-OMeTAD/Ag, and. **e** *J*–*V* curves of the flexible and **f** the large area devices [112]. Copyright © 2022 Wiley–VCH GmbH

Yang et al. used Ti_3_C_2_T_*x*_ MXene nanosheets as a novel kind of ETL in planar structured PSCs that have undergone low-temperature processing [111]. The metallic Ti_3_C_2_T_*x*_ can be improved as an ETL by applying a UV-ozone treatment that boosts the surface Ti–O bonds without affecting the bulk properties, such as high electron mobility. The schematic and cross-sectional SEM image of the PSC device with a configuration of ITO/Ti_3_C_2_T_*x*_/MAPbI_3_/Spiro-OMeTAD/Ag are displayed in Fig. 6a–b, respectively. The thickness of the Ti_3_C_2_T_*x*_ layer is found to be 18 ± 3 nm. Figure 6c shows a schematic illustration of the energy-level diagram for each component of the PSC device. The shift in WF of Ti_3_C_2_T_*x*_ from − 5.52 to − 5.62 eV after UV-ozone treatment enables faster electron transport. The PCE of PSC increased to 5% in the case of Ti_3_C_2_T_*x*_ ETL without UV-ozone treatment and to 17.2% with a Ti_3_C_2_T_*x*_ ETL film after 30 min of UV-ozone treatment because of an improved electron transfer. The UV-ozone treated Ti_3_C_2_T_*x*_ ETL suppressed recombination at the ETL/perovskite interface [111].

Zhang et al. fabricated 2D Nb_2_CT_*x*_ MXene nanosheets ETL and incorporated them into the perovskite absorber layer [112]. The WF of Nb_2_CT_*x*_ was tuned from 4.65 to 4.32 eV by replacing its –F groups with –NH_2_ groups through hydrazine (N_2_H_4_) treatment to match the conduction band minimum of the perovskite layer. Additionally, incorporating N_2_H_4_-treated (T-Nb_2_CT_*x*_) MXene nanosheets with abundant NH_2_ groups slows the crystallization rate of the perovskite precursor by forming a hydrogen bond with iodine ions, which encourages the creation of high-quality and oriented perovskite films. As a result, the PSC with a device configuration of ITO/Nb_2_CT_*x*_/FA_0.85_Cs_0.15_PbI_3_/Spiro-OMeTAD/Ag demonstrated a maximum PCE of 21.79% with T-Nb_2_CT_*x*_ MXene ETLs and T-Nb_2_CT_*x*_ MXene nanosheet additive. The highest PCE values of 19.2% and 18.3% were realized for the corresponding flexible and large-area devices **(**Fig. 6d–f). After 1500 h of storage, the unencapsulated devices still retain 93% of the original PCEs. This research illustrates the wide range of potential applications for 2D Nb_2_CT_*x*_ MXene in photoelectric devices [112].

Despite many benefits of 2D Ti_3_C_2_T_*x*_ MXene, including high transparency, high conductivity, variable WF, and solution processability, the performance of the MXene-based PSC is still subpar to that of the conventional TiO_2_- or SnO_2_-based equivalent. The MXene/perovskite interface has some critical problems that need to be resolved. Wang et al*.* adopted a room-temperature solution technique followed by oxygen plasma treatment to use Ti_3_C_2_T_*x*_ MXene as ETL in PSCs [113]. Oxygen plasma treatment was demonstrated to form abundant Ti*–*O bonds randomly distributed on MXene and disrupt portions of Ti*–*C bonds. In addition to reduced trap states and better electron transport along the interface, the surface change made MXene WFs variable. In addition, contact angle and topography measurements were used to extensively analyze the surface tension of MXene and the related perovskite morphology. The device stability was improved, thanks to the PbO contacts between perovskite and MXene. The champion device attained a high PCE of 18.9% with a steady state output *J*_*sc*_ of 21.5 mA cm^−2^. The unencapsulated PSC with plasma-treated ETL demonstrated long-term stability by retaining 90% of its initial PCE after 750 h of storage in ambient air at 25 °C and RH of ~ 50% [113].

MXenes are appealing for use in PSCs due to their distinct features that result from surface functional groups and oxidation. Yang et al. oxidized Ti_3_C_2_T_*x*_ hydrocolloid to adjust its characteristics for an ETL in low-temperature processed PSCs [114]. The energy levels were calculated using the Vienna ab initio simulation package code, which is based on DFT. Ti_3_C_2_T_*x*_ can be oxidized to produce Ti–O bonds and significantly minimize the macroscopic defects in a spin-coated film. However, after substantial oxidation, the material changes from metallic to semiconductor. In the case of a hybrid of oxidized and pristine Ti_3_C_2_T_*x*_, better matching of energy levels between perovskite and ETL layer results in a champion PCE of 18.3%. The improved electron mobility in the ETL, which encourages electron transport and lowers electron–hole recombination, is responsible for improving the PCE. This work illustrates the significant potential of MXene-derived materials in low-temperature processed PSCs [114].

#### MXenes as Additives in ETLs

**Fig. 7:**
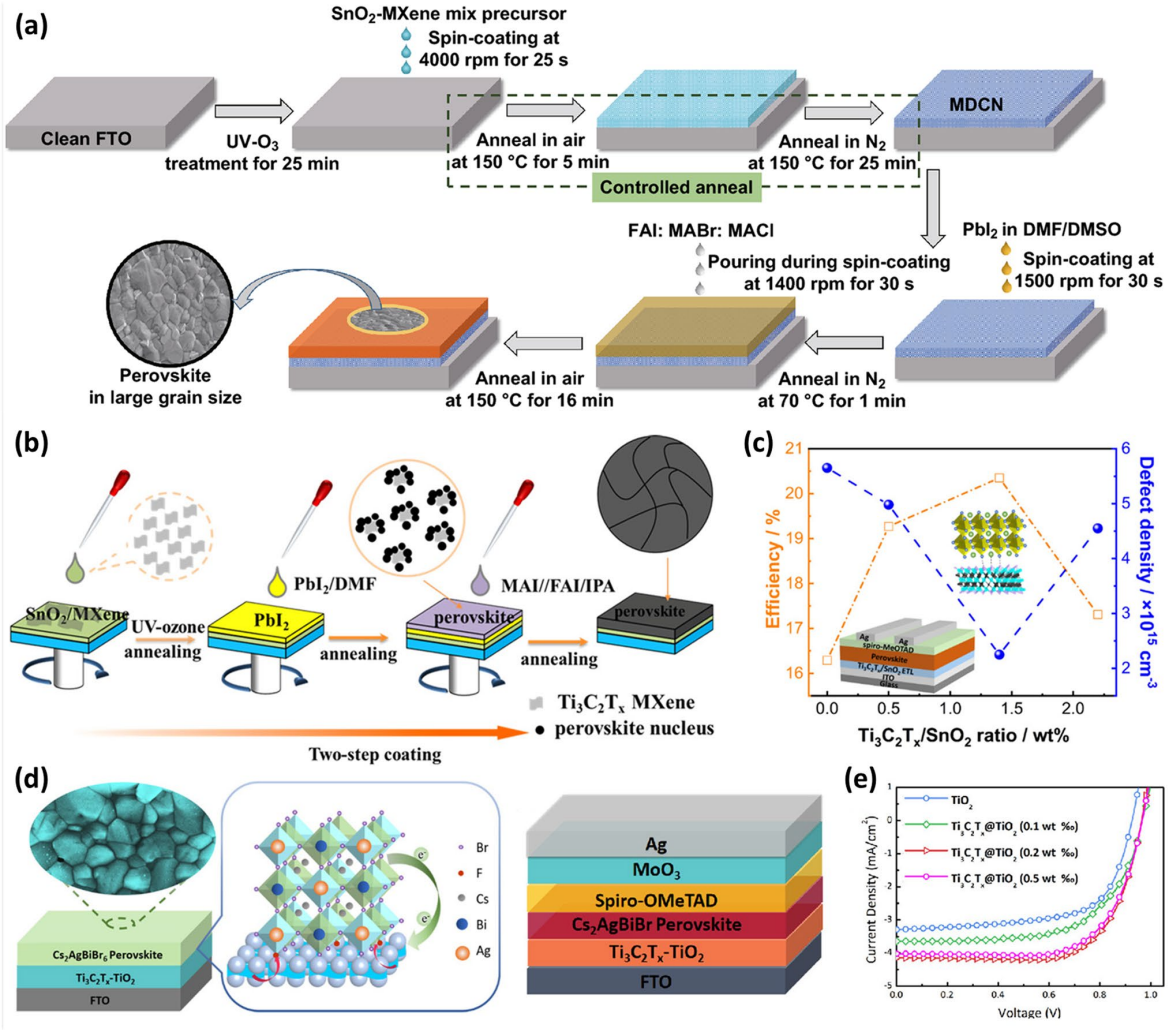
**a** Fabrication process of perovskite layer on MDCN ETL [120]. – Copyright © 2020 Springer. **b** Schematic illustration of the preparation processes of the ETL and the perovskite film. **c** Schematic diagram of PSC and MXene with Perovskite, efficiency, and defect density values with respect to Ti_3_C_2_T_*x*_/SnO_2_ wt% [121]. Copyright © 2021 American Chemical Society. **d** Schematic of Cs_2_AgBiBr_6_/Ti_3_C_2_T_*x*_@TiO_2_ structure with scanning electron microscope (SEM) image illustrating film quality. **e** *J*–*V* curves of planar Cs_2_AgBiBr_6_ PSCs based on TiO_2_ and different weight percentage of Ti_3_C_2_T_*x*_@TiO_2_ [122]. Copyright © 2021 American Chemical Society

SnO_2_ ETLs are extensively used in planar and flexible devices due to a lower annealing temperature (below 185 °C), and better electrical and optical properties [115, 116]. These ETLs have disadvantages of de-wetting property, low transmittance, and conductivity. On the other hand, TiO_2_ ETL is also commonly used for high PSC devices and requires a high annealing temperature of 450–500 °C [117]. Furthermore, the PSC with the SnO_2_ nanoparticle-modified TiO_2_ (SnO_2_@TiO_2_) composite ETL processed at low temperature shows a high PCE of 21.3%. However, the amorphous nature of TiO_2_ at low temperatures limits the PCE of the devices [118, 119]. In 2020, Huang et al. used a multi-dimensional conductive network (MDCN) heterojunction structure made of TiO_2_, SnO_2_, and Ti_3_C_2_T_*x*_ MXene as the electron transport layer to fabricate stable and efficient planar PSCs [120]. Based on an oxygen vacancy scrambling effect, the zero-dimensional anatase TiO_2_ quantum dots are in-situ rooted on three-dimensional SnO_2_ nanoparticles, forming nanoscale TiO_2_/SnO_2_ heterojunctions, and are surrounded by 2D conductive Ti_3_C_2_T_*x*_ sheets. The fabrication process for the (FAPbI_3_)_0.97_(MAPbBr_3_)_0.03_ perovskite thin film with the MDCN ETL is carried out using a controlled low-temperature annealing technique first in an environment of air and later in N_2_ (Fig. 7a). The optical quality, the crystallinity of the perovskite layer, and internal interfaces are all improved by the optimal MXene concentration of 0.02 wt% contributing more carriers with effective and speedy transfer in the device. The resulting PSCs with MDCN ETL-air and N_2_ attained a champion PCE of 19.1%, compared to the PCE of 16.8% for the pristine device with SnO_2_ ETL. Furthermore, the device with MDCN ETL retained nearly 85% of its initial performance for over 45 days in air with a humidity of 30–40%; in contrast, the pristine (control) device only retained about 75% of its initial performance [120].

Defect-free polycrystalline perovskite films are highly desirable for fabricating an effective and stable PSC. However, the use of molecular materials in conventional defect reduction methods suffers from their complicated procedures, poor durability, and limited effects. Zheng et al. used a hybrid film made of SnO_2_ nanoparticles and Ti_3_C_2_T_*x*_ MXene nanoflakes as ETL in a planar regular-structure PSC [121]. The SnO_2_/MXene colloidal suspension is spun onto ITO substrates to form the ETL, and then the perovskite layer (MA_0.15_FA_0.85_PbI_*x*_Br_3-*x*_) is spin-coated on the previously coated ETL via a typical two-step deposition technique (Fig. 7b). The film properties of the top perovskite layers, such as compactness, crystal size, surface roughness, crystallinity, optical absorption, defect density, and so on, are changeable by varying the Ti_3_C_2_T_*x*_/SnO_2_ ratios (0–2.2 wt%) in ETLs. Compared to a pristine device with SnO_2_ ETL, the defect density in perovskite films with an optimized 1.4 wt% hybrid ETL is significantly reduced from 5.65 × 10^15^ to 2.25 × 10^15^ cm^3^. However, the electrical conductivity of the hybrid ETL is decreased, most likely because of the geometric configuration of the added MXene. As a result, the PCE of PSCs with 1.5 wt% Ti_3_C_2_T_*x*_/SnO_2_ ratio is boosted significantly from 16.28 to 20.35%, along with *J*_*sc*_ and FF increasing from 20.65 and 0.71 to 23.65 mA cm^−2^ and 0.76 (Fig. 7c). However, *V*_*oc*_ (1.111 to 1.113 V) remained almost stable. Furthermore, the environmental stability of the unencapsulated devices is dramatically enhanced by retaining 74% of their initial PCE after 768 h of storage under an air environment at 25 °C with an RH of 30% [121].

To address the issue of the instability of the APbX_3_ structure and lead toxicity, the inorganic Cs_2_AgBiBr_6_ double perovskite structure is a *via*ble development route for PSCs. However, the optoelectronic application is severely constrained by the low *J*_*sc*_ and PCE owing to the low crystallization of Cs_2_AgBiBr_6_. Li et al. used a straightforward method to dope single-layered MXene nanosheets into titania (Ti_3_C_2_T_*x*_@TiO_2_) to serve as a versatile ETL for stable and effective Cs_2_AgBiBr_6_ double PSCs [122]. In addition to considerably increasing TiO_2_ electrical conductivity and electron extraction rate, single-layered MXene nanosheets also alter the surface wettability of the electron transport layer and promote the crystallization of Cs_2_AgBiBr_6_ double perovskite in solar cell devices. The Cs_2_AgBiBr_6_ double perovskite films based on both TiO_2_ and Ti_3_C_2_T_*x*_@TiO_2_ ETLs have a similar grain size of 200–500 nm (Fig. 7d–e). The TiO_2_-based Cs_2_AgBiBr_6_ film contains large voids, which are pathways for current leakage. In contrast, the Ti_3_C_2_T_*x*_@TiO_2_-based Cs_2_AgBiBr_6_ film is smooth and void-free. As a result, compared to a device based on TiO_2_ ETL, the PCE increased by more than 40% to 2.8%, and the hysteresis was significantly reduced. Additionally, the Ti_3_C_2_T_*x*_@TiO_2_ device demonstrated long-term operational stability. The Ti_3_C_2_T_*x*_@TiO_2_ device retained 93% of its initial PCE even after 15 days of storage in ambient air [122]. Saranin et al*.* doped MXene in both MAPbI_3_ perovskite absorber layer and PCBM layers in an inverted PSC (glass-ITO/NiO/Perovskite + MXenes/PCBM + MXenes/bathocuproine (BCP)/Ag). The MXene doping improves the band alignment at the perovskite/charge transport layer owing to the WF shift, which facilitates charge extraction at the electrodes. Hence the inverted PSC attained a PCE of 19.20% [91].

To reduce recombination losses and enhance the PCE of PSCs, defect passivation and customizing the perovskite charge transport layer interfaces are crucial. Chava et al. tailored the electrical characteristics of the ETL and the ETL/perovskite interface in inverted (pin) PSCs using Ti_3_C_2_T_*x*_ MXene [123]. A [6, 6]-phenyl-C_61_-butyric acid methyl ester (MPC_61_BM)-based ETL with MXene doping has improved electrical conductivity and band alignment at the ETL/perovskite interface. The n-doping of PC_61_BM was confirmed by a red shift of the A_g_ (2) peak in the Raman spectrum, and a localized upshift of the Fermi level measured using scanning Kelvin probe force microscopy (SKPFM). As a result, PSC devices using M-PC_61_BM as the ETL attained a higher PCE of over 18% than control devices using PC_61_BM as the ETL, with a PCE of 15.55%. Furthermore, the PSC with MXene as an interfacial layer between the perovskite and ETL with a structure of ITO/NiO_*x*_/MXene/MAPbI_3_/PC_61_BM/BCP/Ag attained a maximum PCE of 15.99% with a maximum *V*_*oc*_, *J*_*sc*_, and FF of 1.05 V, 20.74 mA cm^−2^, and 74%, respectively. In comparison, the PSC with a standalone Ti_3_C_2_T_*x*_ MXene ETL showed a maximum of 2.06%, with a maximum *V*_*oc*_, *J*_*sc*_, and FF of 0.72 V, 5.57 mA cm^−2^, and 50%, respectively. The addition of MXene to PSCs has demonstrated diverse effects, including improvement in carrier transport, passivation and trap state reduction, and better interfacial energy alignment [123].

**Fig. 8:**
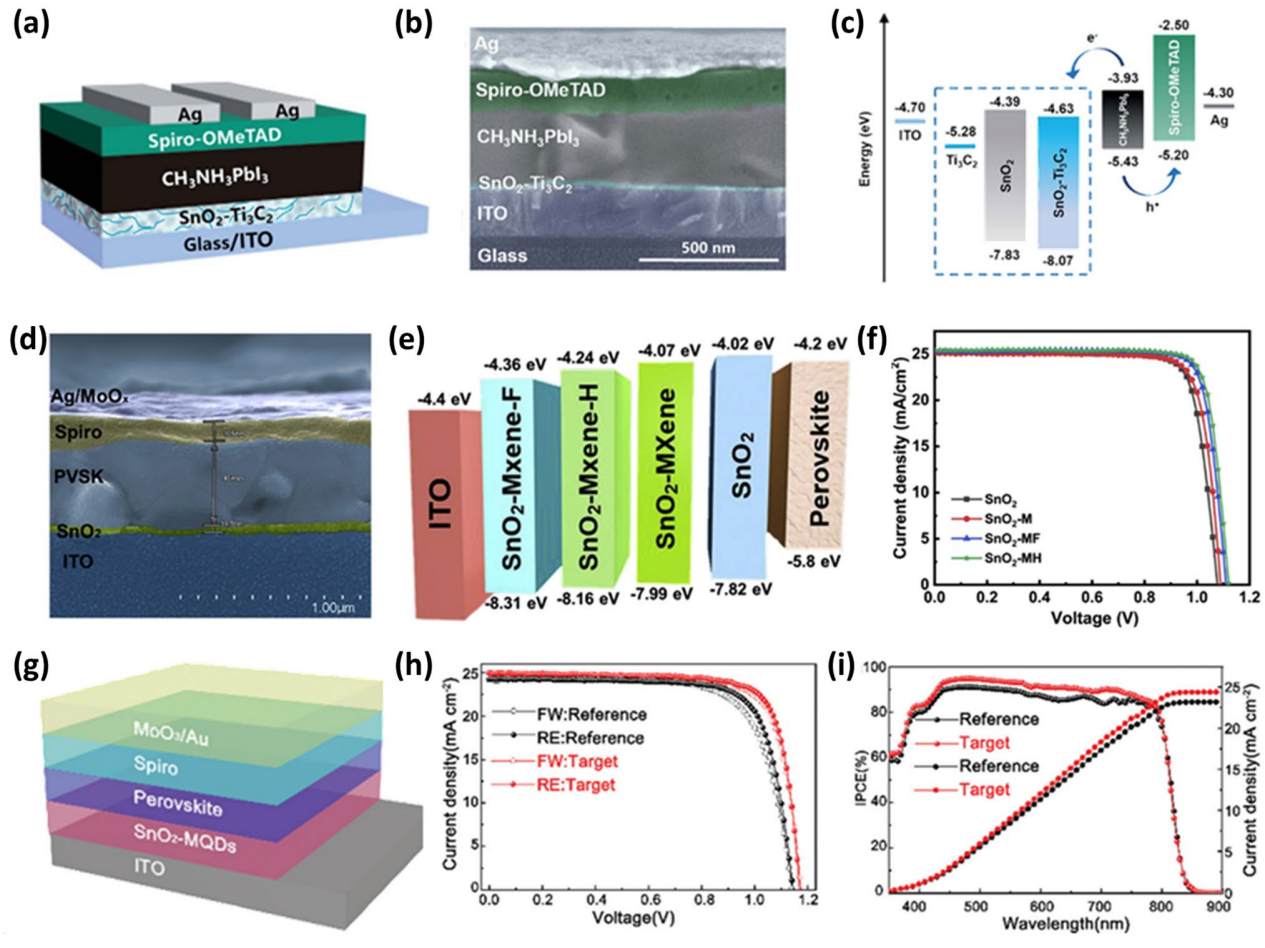
**a** Device architecture of ITO/ETL/MAPbI_3_/Spiro-OMeTAD/Ag based on representative SnO_2_–Ti_3_C_2_ as the ETL. **b** Cross-sectional SEM image of the PSC device. **c** Schematic energy-level diagram of each layer [124]. – Copyright © The Royal Society of Chemistry. **d** Cross-sectional image with ITO/SnO_2_-MH/Perovskite/Spiro/Ag structure. **e** Energy-level diagram of the ITO/ETL/AL structure based on SnO_2_, SnO_2_-M, SnO_2_-MF, and SnO_2_-MH as the ETLs. **f*** J–V* curves of the champion devices based on each ETL under reverse scans for the FAPbI_3_ system [126]. Copyright © 2022 Cell Press. **g** Schematic of the planar heterojunction structure used for the SnO_2_-based PSCs. **h** *J–V* curves of one of the best SnO_2_-based and MQDs-SnO_2_-based PSCs devices under both forward and reverse scans. **i** IPCE curves and integrated current density of these two devices [127]. Copyright © 2021 The Royal Society of Chemistry

Yang et al. utilized SnO_2_-Ti_3_C_2_ MXene (Ti_3_C_2_ Mxene + SnO_2_) nanocomposites with varying Ti_3_C_2_ contents (0, 0.5, 1.0, 2.0, and 2.5 wt%) as ETLs and applied in planar-structured PSCs with an architecture of ITO/SnO_2_ + Ti_3_C_2_T_*x*_/MAPbI_3_/Spiro-OMeTAD/Ag (Fig. 8a) [124]. The corresponding cross-sectional SEM image of the PSC and energy-level diagram of each component are shown in Fig. 8b–c. The lowest occupied molecular orbital reduces from −4.39 eV for pure SnO_2_ ETL to −4.63 eV for SnO_2_ + Ti_3_C_2_T_*x*_ ETL, facilitating fast electron transfer. The PCE of the device fabricated with pure Ti_3_C_2_ as the ETL increases from 17.2 to 18.3% when SnO_2_ and 1.0 wt% Ti_3_C_2_ are combined. The metallic Ti_3_C_2_ MXene nanosheets demonstrated superior charge transfer pathways, improving electron extraction and mobility while lowering electron transfer resistance at the ETL/perovskite interface, resulting in higher photocurrents [124].

ETL materials with good optoelectrical properties and energy levels comparable to the perovskite layer are essential to meet the demand for commercialization. Niu et al. introduced Nb_2_C MXenes as an additive to SnO_2_ ETL for the first time, which caused the SnO_2_ grains to develop clearly [125]. The incorporation of Nb_2_C MXenes leads to an increase in lattice spacing of (101) and (110) planes corresponding to SnO_2_ ETL from 0.32 to 0.33 nm and 0.240 to 0.253 nm, respectively. Further, the roughness and average grain size increased from 3.76 and 179 nm for SnO_2_ ETL to 11.1 and 256 nm for the SnO_2_-Nb_2_C ETL, respectively. In addition, the Nb_2_C inclusion in SnO_2_ also reduces surface energy between the perovskite and the ETL, improving the surface wettability so that the perovskite solution can spread smoothly on the ETL. The light reflection also reduced, indicating improved light absorption and an improved fill factor. The electron mobility for the SnO_2_ ETLs increased from 2.3 × 10^−5^ to 1.39 × 10^−4^ cm^2^ V^−1^ s^−1^ for the SnO_2_-Nb_2_C ETLs, indicating faster electron transfer from perovskites to ETLs. Hence, the PSCs based on the architecture of ITO/SnO_2_-Nb_2_C/Perovskite/Spiro-OMeTAD/Ag exhibited the best PCE of 22.86% (18.96% for the control device) with improved *V*_*oc*_, *J*_*sc*_ and FF. The PSCs with the modified ETL maintain 98% of the original efficiencies after 40 days at 25 °C under a humidity of 40–60% [125].

Yin et al. utilized fluoroalkylsilane and dodecyltrimethoxysilane functionalized MXene nanosheets as dopants into the SnO_2_ ETL to fabricate PSC devices with modified ETL layers, respectively, denoted as SnO_2_-MF and SnO_2_-MH [126]. To fabricate high-performance devices, it is necessary to have an ETL with improved energy alignment and improved charge transfer, which will aid in the efficient extraction and transport of photogenerated carriers. The SnO_2_-MH ETL has improved band alignment, as evidenced by DFT calculations and ultraviolet photoelectron spectra measurements. In the meantime, functionalized MXene nanosheets exhibit strong electrical conductivity and mobility and may quickly and effectively establish a zero Schottky barrier heterojunction with SnO_2_. Finally, the appropriate surface energy attained by functionalized MXene additives can increase the grain size of the perovskite thin film. The schematic of PSC and the energy-level diagram of different SnO_2_, ITO and perovskite layers are sketched in Fig. 8d–e. The devices based on the SnO_2_-MH ETL with the device architecture of ITO/SnO_2_-MH/FAPbI_3_/Spiro/MoO_x_/Ag significantly enhanced their PCE from 21% (with no additive) to 23.66%. The champion device exhibited a maximum PCE of 24.1% with a *J*_*sc*_ of 25.49 mA cm^−2^, a *V*_*oc*_ of 1.121 V, and a FF of 84.4% (Fig. 8f). The PSCs also demonstrated better operational stability and moisture resistance [126].

Yang et al*.* fabricated a modified SnO_2_ bottom layer to produce highly crystalline perovskite films to improve the photovoltaic performance of PSCs [127]. To produce highly crystalline and durable perovskite films, perovskite crystallization mechanisms must be modulated. Ti_3_C_2_T_*x*_ quantum dots (MQDs) were introduced into SnO_2_ ETL to investigate the crystallization kinetics of the perovskite. It was discovered that perovskite nucleation from the precursor solution could be induced quickly by Ti_3_C_2_T_*x*_ MQDs-modified SnO_2_ (MQDs-SnO_2_) ETL, resulting in the formation of an intermediate perovskite phase after anti-solvent treatment. As a result, the perovskite film crystal quality and phase stability are significantly improved. A steady-state PCE of 23.3%, with a *J*_*sc*_ of 24.96 mA cm^−2^, a *V*_*oc*_ of 1.172 V, and an FF of 0.798 were attained for PSCs with a device structure of ITO/SnO_2_-MQD/Perovskite/Spiro/MoO_*x*_/Au (Fig. 8g–h) by taking advantage of the excellent charge extraction capabilities of the MQDs-SnO_2_ layer. The PSC with MQDs-SnO_2_ ETL has higher incident photon-to-electron conversion efficiency (IPCE) over the entire visible region compared to the pristine device. The integrated *J*_*sc*_ value of 24.39 mA cm^−2^ is very close to the observed *J*_*sc*_ value of 24.96 mA cm^−2^ as observed by a solar simulator (Fig. 8i). The fabricated PSC also demonstrated exceptional stability against humidity and light soaking [127].

### MXenes as HTLs or Additives in HTLs

#### MXenes as HTLs

**Fig. 9:**
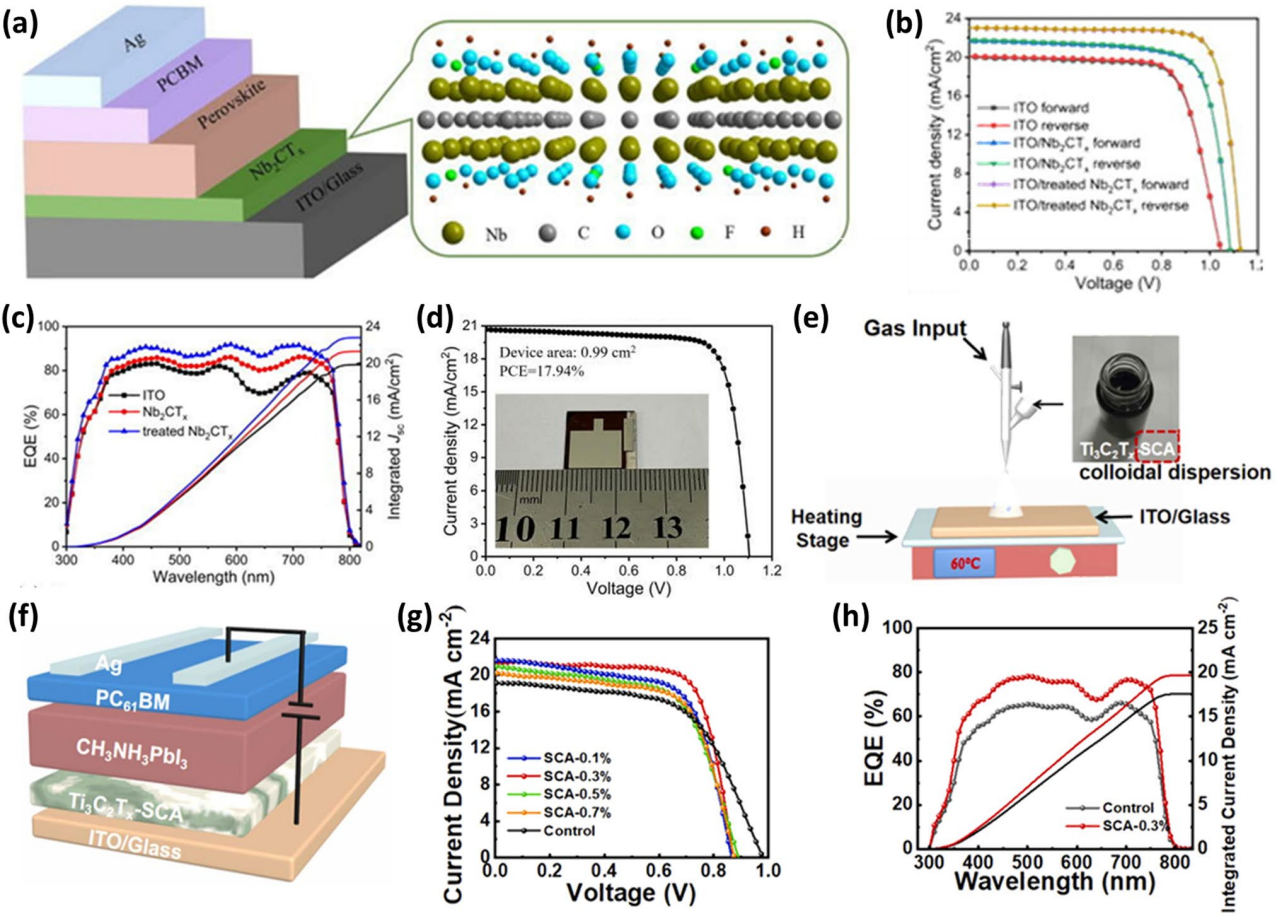
**a** A schematic diagram of the device structure and the structure of Nb_2_CT_*x*_ MXene. **b** *J–V* curves of PVSCs measured under different scan directions. **c** External quantum efficiency (EQE) and integrated *J*_*sc*_ curves of different PSCs. **d** *J–V* curves of the flexible PSCs utilizing oxygen plasma-treated Nb_2_CT_*x*_ HTL [134]. Copyright © 2021 AIP Publishing. **e** Schematic representation of spray coating process to form the Ti_3_C_2_T_*x*_-SCA film and structural formula of vinyl tris (2-methoxy ethoxy) silane. **f** Schematic diagram of a device with ITO/HTL/Perovskite/PC_61_BM/Ag structure. **g** *J–V* curve of PSCs based on Ti_3_C_2_T_*x*_, with different volume fractions of SCA as HTL. **h** EQE spectrum and corresponding integrated current density [139]. Copyright © 2022 Elsevier

In PSCs, the HTL plays a vital role in controlling the crystallization of a perovskite film and the hole transfer efficiency at the perovskite/HTL interface. Although many successes in PSC by organic hole transport materials (HTMs) such as N,N’-bis(3-methylphenyl)-N,N’-bis(phenyl) benzidine, poly(trimethylene terephthalate-co-trimethylene isophthalate)-Terephthalic acid (PTTI-TPA), triphenylamine-based HTM incorporating pyridine core (coded as H-Pyr) and pyridine-based polymer semiconductor (PPY2), the organic HTLs fail to scale up the mass-production due to complexity of synthesis, purification and the manufacturing cost associated with the synthesis procedures [128–130]. In contrast, the common inorganic HTM NiO_*x*_ requires an annealing temperature above 200 °C and is unsuitable for flexible devices. Hence, low-cost and low-temperature processed HTMs with excellent electrical properties are necessary. Nb_2_CT_*x*_ MXene is a promising HTM due to good conductivity and an adjustable WF [131, 132]. Furthermore, the Nb_2_CT_*x*_ MXene has three atomic layers compared to the five atomic layers of the Ti_3_C_2_T_*x*_ MXene (excluding atomic layers of surface functional groups). Hence Nb_2_CT_*x*_ has a larger specific area, and consequently, the contact area with the perovskite layer is also larger. This promptly increased the charge transfer in PSCs. The charge transfer depends upon the WF of Nb_2_CT_*x*_ MXene. However, the WF can be controlled depending on the type and quantity of the surface-terminated functional groups [59, 133]. As observed from First-principles calculations, the WF of the pristine Nb_2_CT_*x*_ nanosheets with –O, –OH, and –F surface functional groups is approximately 4.7 eV, which is not enough to transport holes across the Nb_2_CT_*x*_/MAPbI_3_ interface. Commonly, O-terminated Nb_2_CT_*x*_ shows a higher WF compared to the F- and OH-terminated counterparts. This WF difference arises due to the different surface dipole moments and a difference in charge transfer between the terminated functional groups and Nb atoms [59, 61]. In 2021, Zhang et al. synthesized the highly photoelectric Nb_2_CT_*x*_ MXene by oxygen plasma treatment and used it as the HTL in inverted PSCs [134]. The oxygen plasma-treated Nb_2_CT_*x*_ MXene nanosheets showed enhanced conductivity and high transmittance. The oxygen plasma treatment modified the WF of Nb_2_CT_*x*_ from 4.68 eV (pristine) to 5.04 eV by enhancing the O functional groups on the Nb_2_CT_*x*_ surface. In other words, the high electronegative O atoms pull electrons from Nb atoms, thus reducing the Fermi level of Nb_2_CT_*x*_. At the same time, the O functional groups induce a dipole moment with O atoms facing toward Nb atoms, uplifting the vacuum level. The combined effect of the downshifted Fermi level and uplifted vacuum level increased the WF of the Nb_2_CT_*x*_. This high WF reduced energy offset/loss at the interface between the HTL/perovskite absorber layer and eased the hole transfer from the perovskite layer to HTL, leading to suppressed charge recombination and high *J*_*sc*_. Furthermore, the photoluminescence (PL) spectra from the perovskite film with the oxygen plasma treated Nb_2_CT_*x*_ (Nb_2_CT_*x*_/MAPbI_3_) indicated the most significant PL intensity quenching, suggesting an improved hole extraction ability of Nb_2_CT_*x*_. From time-resolved PL spectra, the average PL lifetime is found to decrease from 16.51 ns (glass/MAPbI_3_) to 2.26 ns (ITO/MAPbI_3_), 1.64 ns (ITO/Nb_2_CT_*x*_/MAPbI_3_), and 0.72 ns (ITO/treated Nb_2_CT_*x*_/MAPbI_3_), which further confirms the excellent hole transportability of Nb_2_CT_*x*_. The PSC with the plasma-treated Nb_2_CT_*x*_ MXene HTL with a device structure of ITO/Nb_2_CT_*x*_/MAPbI_3_/PCBM/Ag exhibits a higher PCE of 20.74% with increased *V*_*oc*_ and *J*_*sc*_ than those with the pristine Nb_2_CT_*x*_ (18.1%) and without Nb_2_CT_*x*_ MXene HTLs (15.5%). The higher external quantum efficiency (EQE) values indicate the enhanced* J*_*sc*_, suggesting more efficient charge separation and collection efficiency. The PSCs with oxygen plasma-treated Nb_2_CT_*x*_ HTLs attain the highest EQE and integrated *J*_*sc*_ (22.75 mA cm^−2^), close to the *J*_*sc*_ values obtained from the *J–V* measurements (Fig. 9c). Additionally, the flexible and large area (0.99 cm^2^) PSCs with oxygen plasma treated Nb_2_CT_*x*_ HTLs exhibit the highest PCE of 17.3% and 17.9%, respectively. Moreover, the Nb_2_CT_*x*_ HTL-based PSCs show excellent long-term stability in the glovebox for 70 days and thermal stability at 85 °C [134].

A silane coupling agent (SCA) can connect various materials and enhance the interface properties [135, 136]. Furthermore, this coupling agent reduces the cost by increasing the corrosion resistance of the material [137, 138]. However, SCA is hard to use directly for bonding. Including hydroxyl groups in SCA is a possibility to improve the interface bonding. In 2022, Du et al. fabricated an HTL by spraying Ti_3_C_2_T_*x*_ MXene nanosheets with varying concentrations of the SCA (vinyltris (2-methoxyethoxylsilane)) (0, 0.1, 0.3, 0.5, and 0.7 vol%) [139]. As shown in the schematic of the spray deposition device (Fig. 9e), the ITO glass substrate is placed on the 60 °C hot stage, and the nozzle of the spray gun is set 20 cm above. The Ti_3_C_2_T_*x*_-SCA dispersion of different proportions was deposited uniformly on the ITO substrate, setting the spray speed of the spray gun to 2 mL min^−1^_._ A flat, pin-hole-free Ti_3_C_2_T_*x*_ film was produced with a mixing content of 0.3 V/V% by utilizing SCA to efficiently adjust the interface distribution of Ti_3_C_2_T_*x*_ after film fabrication. The superior production of Ti_3_C_2_T_*x*_ films enabled better hole transfer paths, improved hole extraction (hole mobility increases from 1.30 × 10^−6^ to 3.08 × 10^−7^ cm^2^ V^−1^ s^−1^), and lower transfer resistance from 369.3 Ω to 194.1 Ω at the HTL/perovskite interface. The compared values are for the Ti_3_C_2_T_*x*_ HTL and Ti_3_C_2_T_*x*_ + 0.3% SCA HTL. As a result, the device with the structure of ITO/ Ti_3_C_2_T_*x*_-0.3SCA/MAPbI_3_/PC_61_BM/Ag exhibited an increased PCE from 11.1% (pristine Ti_3_C_2_T_*x*_) to 13.7%. The schematic of the PSC and the *J–V* curves of PSCs with Ti_3_C_2_T_*x*_-SCA, having different concentrations of SCA, are shown in Fig. 9f–g. However, the *V*_*oc*_ dropped by 11%. The voltage drop is attributed to stacking due to high surface roughness (root-mean-square (RMS) = 0.87 nm for only nanosheet to 4.07 nm for nanosheet and SCA) and good coverage of Ti_3_C_2_T_*x*_ [38, 39]. Additionally, the calculated EQE (17.75 and 19.94 mA cm^−2^.) are well consistent with the observed *J*_*sc*_ variations (19.19 mA cm^−2^ and 21.30 mA cm^−2^) for the PSCs with or without SCA (Fig. 9h). Moreover, the PSC based on Ti_3_C_2_T_*x*_-SCA HTL exhibited excellent stability at ambient air humidity ~ 20% and room temperature, and retained about 80% of the initial PCE after 80 h of storage [139].

#### MXenes as Additives in HTLs

As stated earlier, apart from the complexity of synthesis and purification, the poor conductivity, low carrier mobility, resistance to processing solvents, and low transmittance in the UV–vis range of the inorganic HTMs such as CuO_*x*_, Fe_2_O_3_, CuSCN, and NiO_*x*_ and the organic-based polymers poly (3-hexylthiophene) P3HT, poly(triaryl amine) (PTAA), modified fluorene–dithiophene (FDT), and poly[2,5-bis(2-decyldodecyl)-pyrrolo[3,4-c]pyrrole-1,4(2H,5H)-dione-(E)-1,2-di(2,2′-bithiophen-5-yl)-ethene] (PDPPDBTE) limit the commercial applications as HTLs in PSCs [140–146]. Solution-processed poly (3,4-ethylene dioxythiophene):poly- (styrenesulfonate) (PEDOT:PSS) is used as a common HTL due to its high conductivity, appropriate WF, and high transparency. However, its acidic and hygroscopic nature leads to a low efficiency and stability of PSCs [147].

**Fig. 10:**
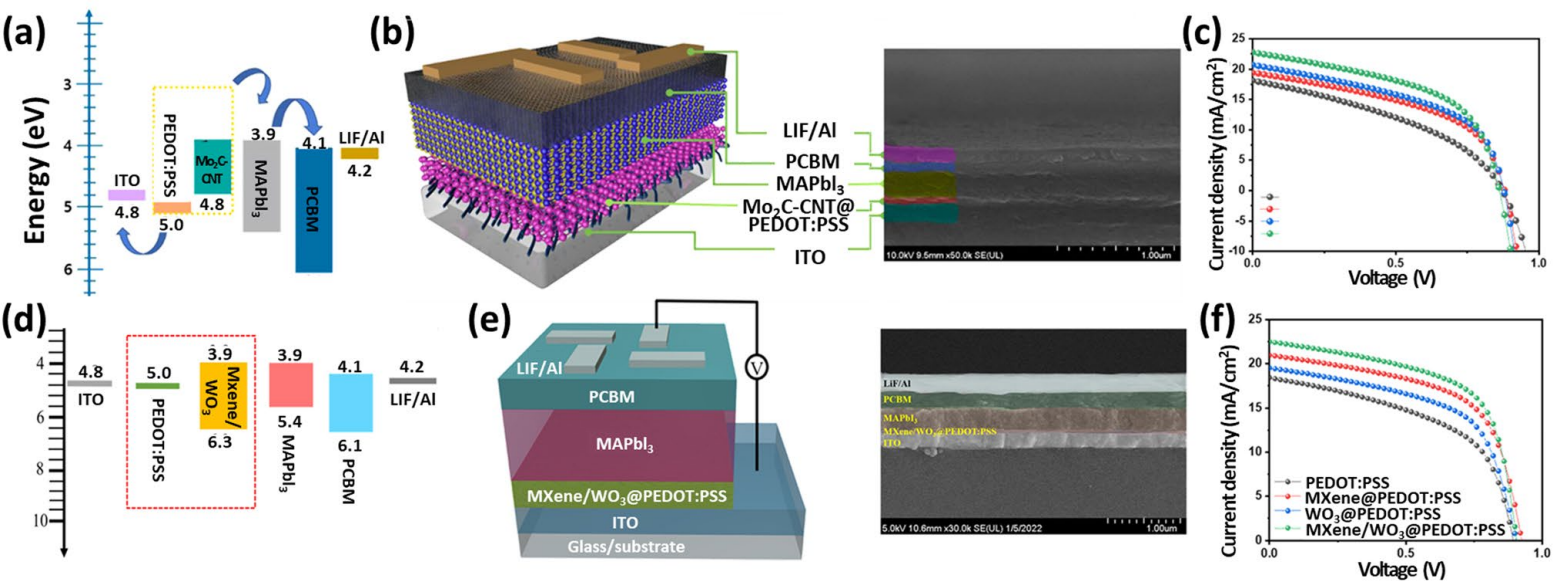
**a** Energy-level diagram for ITO/Mo_2_C-CNTs@PEDOT:PSS/MAPbI_3_/PCBM/LiF/Al structure. **b** Schematic of the fabricated PSC with a device architecture of ITO/ITO/Mo_2_C-CNTs@PEDOT:PSS/MAPbI_3_/PCBM/LiF/Al and its cross-sectional field-emission scanning electron microscopy (FE-SEM) image. **c** *J–V* characteristics of prepared PSC devices using pure and 1.5 wt% of Mo_2_C, CNTs, and Mo_2_C-CNTs blended PEDOT:PSS HTLs [153]. Copyright © 2021 Elsevier.** d** Energy-level diagram, **e** schematic view, and cross-sectional FE-SEM image of the ITO/MXene/WO_3_@PEDOT:PSS HTL/MAPbI_3_/PCBM/LiF/Al prototype structure. **f** *J–V* profiles of PSCs with pure and 2 wt% of WO_3_, MXene, and MXene/WO_3_ doped HTL [154]. Copyright © 2022 Wiley

In contrast, transition metal carbide (Mo_2_C) and carbon nanotubes (CNTs) are promising HTL candidates owing to their high metallic conductivity, higher charge extraction probability, and tunable WF [148–152]. Hussain et al. combined Mo_2_C and CNTs to form a conductive Mo_2_C-CNT hybrid network for the first time with a PEDOT:PSS HTL in PSCs with a device configuration of ITO/Mo_2_C-CNT@PEDOT:PSS/MAPbI_3_/PCBM/LiF [153]. As seen from the energy-level diagram in Fig. 10a, the absorbed light produces electron–hole pairs as the light is incident on the active layer. The pairs get separated and move toward the ETL and HTL. The modified PEDOT:PSS with Mo_2_C-CNT act as exciton dissociation centers and facilitate fast charge separation between MAPbI_3_ and ITO and fast charge transfer to the anode. The schematic of the fabricated PSC with the modified HTL, 1.5 wt% Mo_2_C-CNT@PEDOT:PSS HTL, of PEDOT:PSS and Mo_2_C-CNT is shown in Fig. 10b. The PSC with the modified HTL exhibits the highest PCE of 12% compared to 9.2% (Pure PEDOT:PSS), 9.82% (Mo_2_C@PEDOPT:PSS) and 10.61% (CNT@PEDOPT:PSS) under AM 1.5 G illumination at 100 mW cm^−2^ (Fig. 10c). Additionally, the pure and Mo_2_C, CNT, and Mo_2_C-CNT blended PEDOT:PSS HTLs showed a conductivity of 410.12, 581.73, 604.25, and 712.34 S cm^−1^, respectively, thus evidencing high charge carrier collection/extraction and low internal resistance [148]. In further work, Hussain et al. decorated WO_3_ nanoparticles on 2D conductive Ti_3_C_2_T_*x*_ MXene sheets to fabricate an MXene/WO_3_ hybrid structure and then blended HTL with PEDOT:PSS for PSCs using a simple solution process [154]. The n-type WO_3_ semiconducting material is a promising HTL candidate owing to high electron mobilities (10–20 cm^2^ V^−1^ s^−1^), a tunable bandgap (2.7–3.9 eV), inexpensiveness, high stability against moisture, and possible fabrication at room temperature [6]. On the other hand, Ti_3_C_2_T_*x*_ MXene possesses high electrical conductivity (2 × 10^4^ S cm^−1^) and high mobility of 1 cm^2^ V^−1^ s^−1^ [28, 30]. An MXene/WO_3_ hybrid structure with 1, 2, and 3 wt% was used with PEDOT:PSS to change the perovskite hybrid module's HTL for a highly efficient planar solar cell with a configuration of glass/ITO/MXene/WO_3_@PEDOPT:PSS/MAPbI_3_/PCBM/LiF/Al. The corresponding energy band diagram and schematic are shown in Fig. 10d–e. The energy band level of MXene/WO_3_ is well matched with the perovskite active layer and forms good interfacial contact. The fabricated perovskite solar cell using the optimal device configuration of 2 wt% MXene/WO_3_/PEDOT:PSS HTL achieves the highest PCE of 12.26 ± 0.12%. The PCEs of PSCs with pure PEDOT:PSS, WO_3_/PEDOT:PSS, and WO_3_/PEDOT:PSS are 9.19 ± 0.12%, 10.13 ± 0.14% and 11.42 ± 0.13%, respectively. Furthermore, the PSCs with pure MXene, WO_3_, and MXene/WO_3_ nanostructure as HTLs show low PCEs of 6.51%, 6.68%, and 6.87%, respectively. Additionally, the RMS surface roughness of the MXene/WO_3_ composite with PEDOT:PSS decreases to 21.71 nm for WO_3_ (35.79 nm) and MXene (34.23 nm) blended PEDOT:PSS, indicating better adaptable surface behavior with finely dispersed nanoparticles and reduced domain sizes, which results in enhanced interfaced characteristics. The MXene/WO_3_ heterostructure also showed high crystallinity, low shunt resistance ~ 5004 ± 68 Ω cm^2^_,_ and series resistance ~ 157 ± 2 Ω cm^2^. These results suggest better exciton separation and passivation of trapping centers by the densely blended smooth MXene/WO_3_ thin films. These blended nanostructures efficiently tune the HTL/perovskite interface through their active interface for charge transfer and collection and suitably alter energy band alignment, resulting in facile charge extraction and the shortest charge carrier lifetime [154].

### MXenes as Electrodes or Additives in Electrodes

**Fig. 11:**
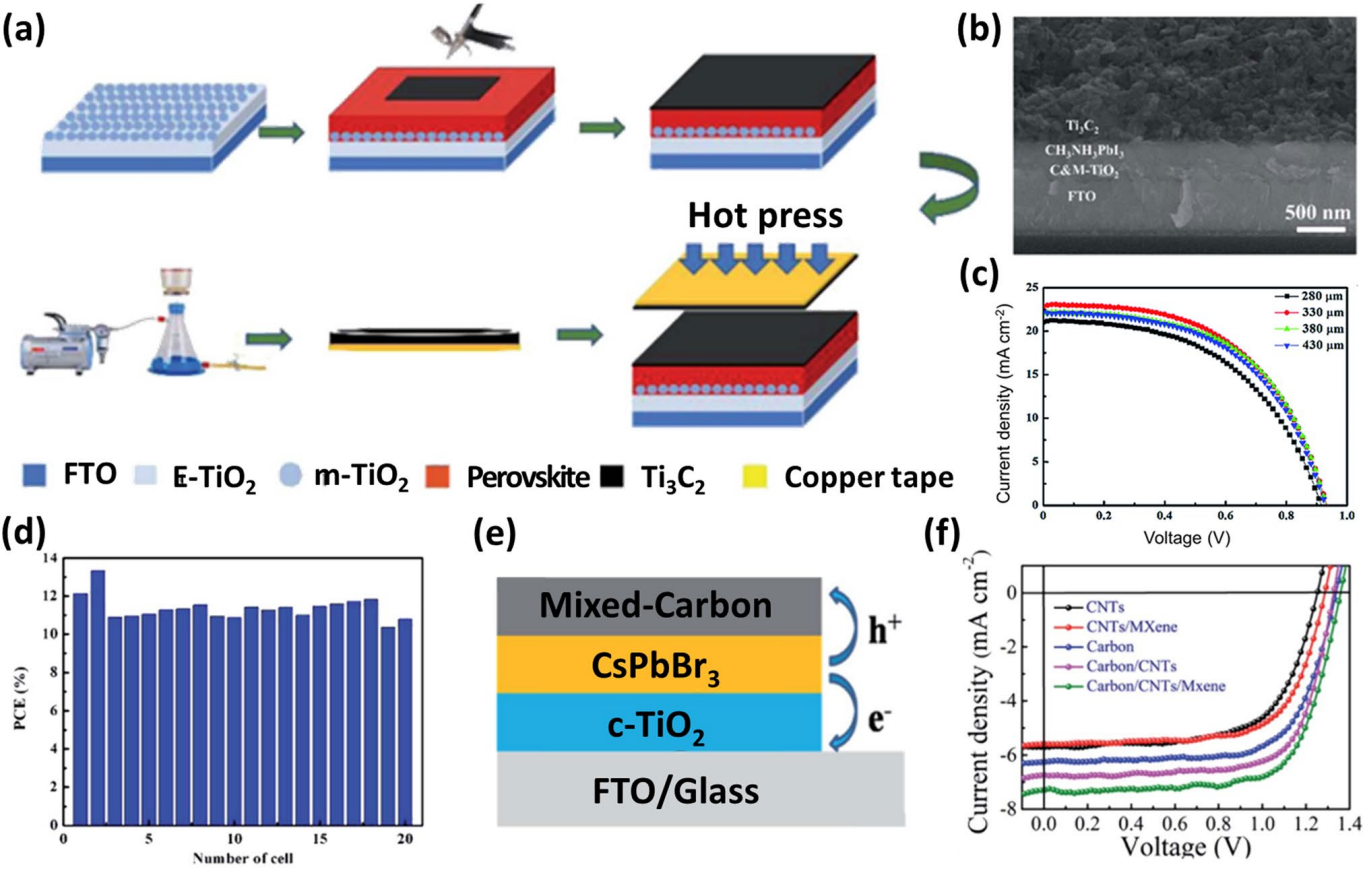
**a** Schematic diagram showing the fabrication process of the Ti_3_C_2_ electrode by the hot-pressing method. **b** Cross-sectional SEM image of the PSC based on Ti_3_C_2_ electrode. **c** *J–V* curves of devices with different thicknesses of the Ti_3_C electrode. **d** PCE histogram of the PSCs obtained from the measurements of 20 devices [157]. Copyright © 2019 The Royal Society of Chemistry. **e** Schematic structure of the CsPbBr_3_ solar cell with mixed carbon electrode. **f** *J–V* curves of devices with different types of electrodes [161]. Copyright © 2020 The Royal Society of Chemistry

The large-scale commercialization of PSCs is constrained by the high cost of commonly used HTM—(Spiro-OMeTAD, PTAA, NiO_x_, etc.) and noble metal electrodes (Au and Ag) [155, 156]. Low-cost coal-based carbon electrodes are considered reasonable replacements for both the HTMs and the noble metal electrodes in PSCs. However, the PCEs of PSCs with carbon electrodes are still lower than those of conventional devices. In 2019, Cao et al. were the first to use a more conductive 2D MXene material (Ti_3_C_2_) with an energy level comparable to carbon materials as back electrodes in HTM and noble-metal-free MAPbI_3_ PSCs [157]. Using a straightforward hot-pressing technique at 85 °C and 0.4 MPa, a seamless interfacial contact between the MAPbI_3_ perovskite layer and Ti_3_C_2_ material was achieved, as seen in Fig. 11a–b. The WF of the Ti_3_C_2_ material was found to be 4.96 eV and matches well with the valence band (5.4 eV) of the MAPbI_3_ layer. The hole charge carriers easily transfer from the perovskite layer to the Ti_3_C_2_ electrode and the electrons from the TiO_2_ layer to the FTO electrode. Furthermore, the square resistance decreased from 30.93 to 25.34 Ω sq^−1^ as the thickness of the Ti_3_C_2_ electrodes increased from 280 to 330 mm. The Nyquist plot measured under the illumination of 100 mW cm^−2^ at a bias voltage of 0.60 V and a frequency range of 100 mHz to 1 MHz shows a similar series and decreased charge transport resistance. The decreased charge transport resistance means efficient hole extraction. However, as the thickness of the electrode increases further, transport resistance increases, and hence the PSC shows low photovoltaic performance. Because of the better conductivity and favorable interfacial contact between the optimally thick Ti_3_C_2_ electrode and the perovskite layer, the champion PSC based on this electrode with the device structure of FTO/TiO_2_/MAPbI_3_/Ti_3_C_2_T_*x*_ exhibited a PCE of 13.83% with a *V*_*oc*_ of 0.95 V, a* J*_*sc*_ of 22.97 mA cm^−2^, and an FF of 63% (Fig. 11c). The resulting PCE value is 27% higher than that of the control PSC (10.87%) based on carbon electrodes. A good reproducibility in terms of manufacturability without significant batch-to-batch variations was also observed in the fabrication of PSCs based on Ti_3_C_2_ electrodes (Fig. 11d). The device also demonstrated greater stability than the conventional FTO/compact (c) and mesoporous (m) -TiO_2_/Perovskite/Spiro-OMeTAD/Au device for 360 h when stored at room temperature at an RH of 30%. The Ti_3_C_2_ electrode plays the role of an encapsulating layer, preventing the active or absorber layer from reacting with air and water [157].

As stated earlier, the high costs of HTMs and noble metals impede the scaling of commercialization of PSCs. The straightforward manufacturing processes, low cost, and great stability of carbon-based inorganic PSCs have shown excellent performance in photovoltaics. Although devices with carbon electrodes are less efficient than those with traditional structures, interest in their potential large-scale applications has grown. However, the commercially available carbon paste forms a point contact with the perovskite layer because of the point-to-point contact in the carbon electrode. As a result, many pinholes remain at the interface between the electrode and the perovskite layer, impeding the carrier transport [158]. CNTs with a one-dimensional (1D) structure show high conductivity and direct transfer paths for charge carriers, and 2D Ti_3_C_2_T_*x*_ MXene has high surface areas and shows high conductivity via accelerating the charge carriers [31, 124, 159, 160]. In 2020, Mi et al. incorporated commercial CNTs and 2D Ti_3_C_2_ MXene into the carbon paste and formed a mixed carbon electrode for the inorganic CsPbBr_3_ perovskite-based PSC [161]. This mixed carbon electrode offers a network structure and a multi-dimensional charge transfer path due to a good interface with the perovskite absorber layer. MXenes nanosheets fill the voids of carbon powders. This synergistic structure significantly boosts the conductivity of the carbon electrode and carrier transport. The pure carbon electrode only shows point-to-point contact, and the device with the structure of FTO/c-TiO_2_/CsPbBr_3_/pure carbon exhibits a PCE of 5.9%. For the inorganic CsPbBr_3_ PSC with the mixed carbon electrode, the champion device with the configuration of FTO/c-TiO_2_/CsPbBr_3_/mixed carbon achieves a decent PCE of 7.09% with a *V*_*oc*_ of 1.357 V, a* J*_*sc*_ of 7.16 mA cm^−2^, and an FF of 72.97% (Fig. 11e–f). Furthermore, the devices with the mixed carbon electrodes show good reproducibility and excellent stability by retaining 80% of their initial PCEs after storage in air for one month [161].

**Fig. 12:**
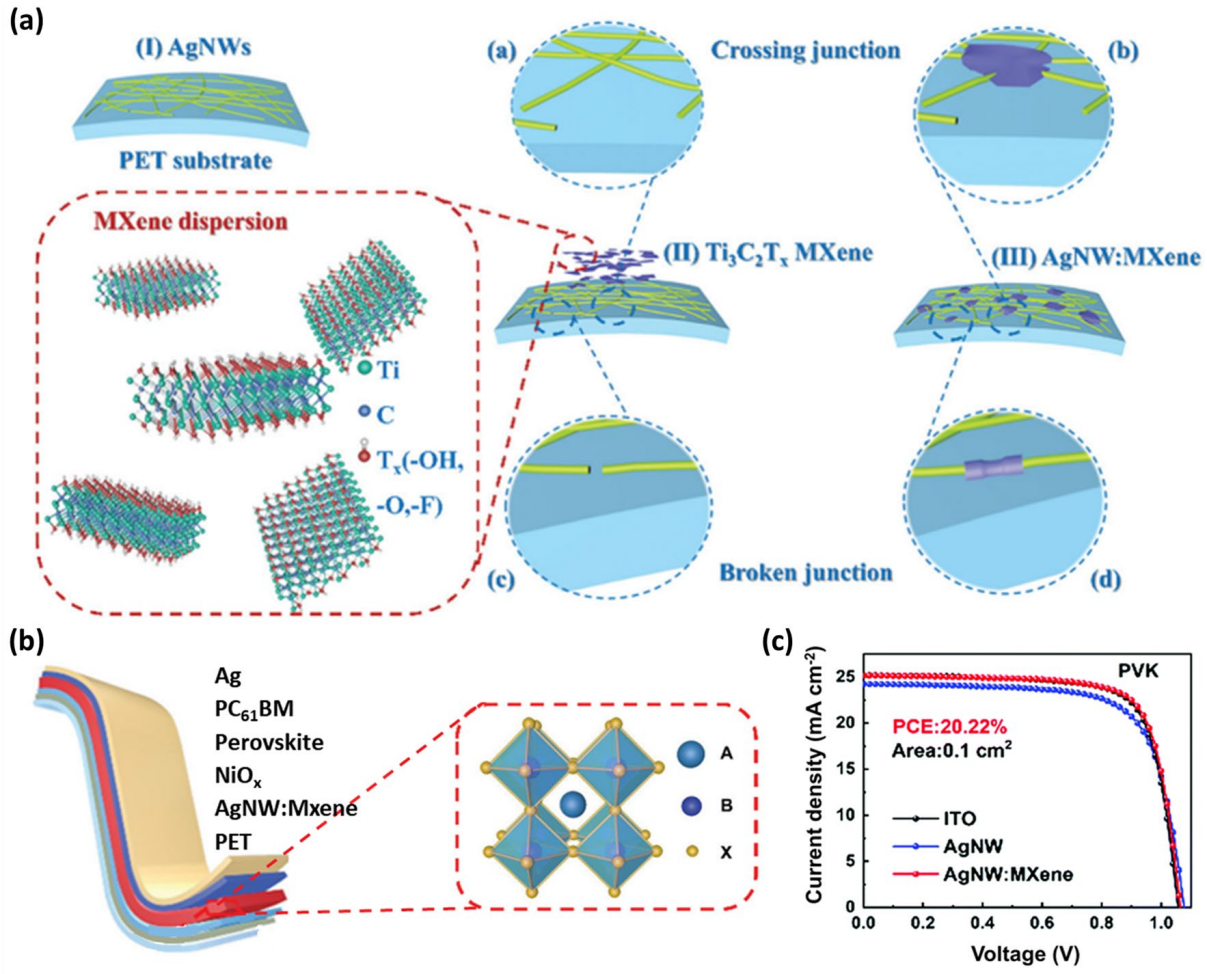
**a** Schematic of the Ti_3_C_2_T_*x*_ MXene and the fabrication procedure of AgNW:MXene flexible transparent conductive electrodes. **b** Flexible PSCs with the structure of PET/AgNW:MXene/NiO_x_/Perovskite/PC_61_BM/Ag. **c** *J–V* curves of the flexible PSCs based on ITO, AgNW, and AgNW:MXene electrodes [176]. Copyright © 2022 The Royal Society of Chemistry

Wearable and other flexible optoelectronic systems require flexible transparent electrodes (FTEs). The excellent conductivity and transparency properties of ITO have placed it as the most reported transparent electrode material among rigid photoelectric devices [162–165]. However, poor mechanical stability (brittleness) and expensive manufacturing equipment costs of ITO electrodes prevent them from being used in flexible electronics. Silver nanowires (AgNWs) have low sheet resistance and high transmittance and are solution-processable [166–168]. They also show super-bending resistance and mechanical stability compared to ITO electrodes. However, AgNWs suffer from high roughness and low adhesion with the substrate and are prone to oxidation and high wire-wire junction resistance [169, 170]. Metal oxide nanoparticles and 2D materials such as electrochemically exfoliated graphene, graphene oxide, and reduced graphene oxide can modify the conductivity and roughness of AgNW networks [171–173]. However, poor doping stability hinders the performance of graphene-based devices [174]. Ti_3_C_2_T_*x*_ MXene, a 2D material, can enhance the performance of AgNW networks due to its high electrical conductivity, carrier mobility, tunable WF, and superior mechanical properties [126, 175]. In 2022, Chen et al. combined 1D AgNWs and 2D Ti_3_C_2_T_*x*_ MXene nanosheets to fabricate composite Ti_3_C_2_T_*x*_ MXene FTEs for flexible solar cell devices [176]. Under electrostatic interaction, a composite 1D:2D structure (AgNW:MXene) FTE is produced. The 1D AgNWs deposited on hydrophilic PET substrates are coated with the MXene nanosheets to form a conductive AgNW:MXene composite network, as shown in Fig. 12a. The MXene nanosheets weld the crossing junctions and the broken junctions. Using capillary force, 2D MXene nanosheets fill the gaps in the AgNW networks and join the wire-wire connections. These provide more continuous conductive paths, improving the conductivity of the electrodes. Additionally, the oxygen-containing functional groups of MXene nanosheets adhere strongly to the PET substrate via a strong hydrogen bonding interaction. The photoelectric performance of FTEs is determined by the figure of merit (FoM). The FTE fabricated with 5 mg mL^−1^ AgNWs dispersion deposited with a concentration of 0.5 mg mL^−1^ MXene shows an FoM by a low sheet resistance of 10.91 Ω sq^−1^ (12.09 Ω sq^−1^ for AgNW without MXene) and a high transmittance of 82.84% (81.96%) at 550 nm. The roughness of the AgNW: MXene FTEs is also decreased, as observed from the RMS roughness of AgNW: MXene FTEs (13.6 nm) compared to the pristine AgNW FTEs (19.2 nm). The mechanical endurance of FTEs determines the ability of FTE to sustain a continuous conductive path to collect charge under an external mechanical stress [177]. The PET/AgNW: MXene shows a surface Young’s modulus of 0.78 GPa, compared to 0.96 and 1.15 GPa of PET/ITO and PET/AgNW, respectively. Additionally, the AgNW:MXene FTEs exhibit superior long-term stability at 60% RH and 120 °C for 240 h, as well as strong mechanical resilience after 1,000 cycles of bending tests at a 5 mm curvature radius. Furthermore, WF matching is important for accelerating the charge transfer. The WF of AgNW:MXene is calculated to be 4.81 eV, which lies in the required normal range for optoelectronic devices. As a result, the inverted PSC with a device structure of PET/AgNW:MXene/NiO_*x*_/Perovskite/PC_61_BM/Ag (Fig. 12b) and an area of 0.1 cm^2^ show a high PCE of 20.22% (18.7% for the cell with pristine AgNWs only) with *V*_*oc*_ of 1.06 V, *J*_*sc*_ of 25.16 mA cm^−2^_,_ and FF of 75.5% (Fig. 12c) [176].

## MXenes as Interfacial Layers

### At the Interfaces Between Perovskites and Electrodes

**Fig. 13:**
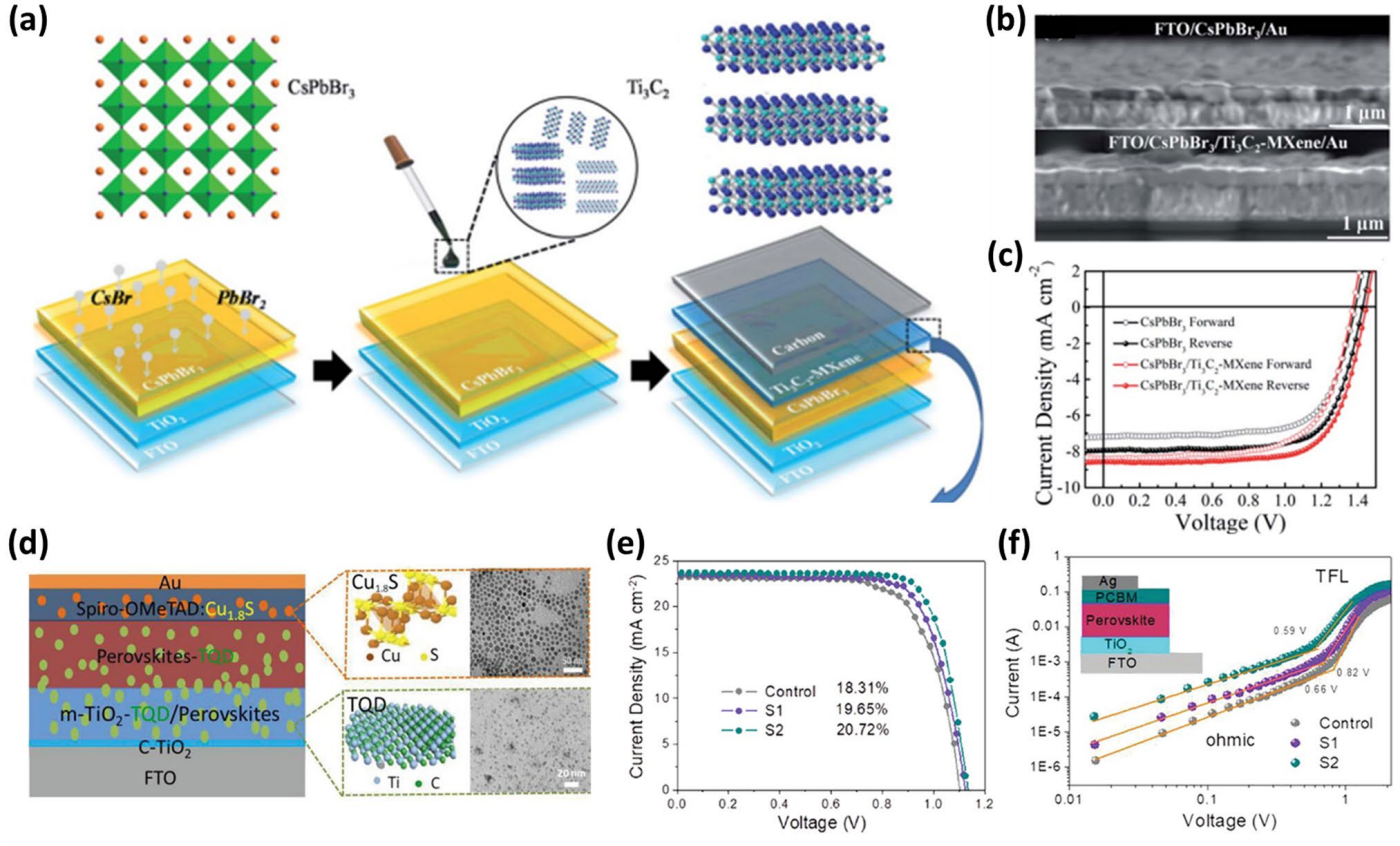
**a** Crystal structure diagrams of CsPbBr_3_ and Ti_3_C_2_ MXene, and schematic for the fabrication of the CsPbBr_3_/Ti_3_C_2_ MXene-based solar cell. **b** Cross-sectional SEM images of FTO/CsPbBr_3_/Au devices with/without Ti_3_C_2_ MXene. **c** *J–V* curves of devices with/without Ti_3_C_2_ MXene [160]. Copyright © 2019 The Royal Society of Chemistry.** d** Architecture of the perovskite solar cells, schematic structure and TEM images of Cu_1.8_S and Ti_3_C_2_ QDs, and the cross-sectional SEM of a complete device. **e** *J–V* curves for FTO/TiO_2_/perovskite, FTO/TiO_2_/MQD/perovskite, and FTO/TiO_2_/MQD/MQD-perovskite devices measured under simulated AM 1.5 sunlight of 100 mW cm^−2^ irradiance. **f** Double logarithmic *J–V* characteristics in electron-only devices with the structure of FTO/TiO_2_/Perovskite/PCBM/Ag (control), FTO/TiO_2_/TQD/Perovskite/PCBM/Ag (S1), and FTO/TiO_2_/TQD-Perovskite/PCBM/Ag (S2) devices [180]. Copyright © 2020 WILEY–VCH Verlag GmbH & Co. KGaA, Weinheim

MXenes are promising candidates in photovoltaics because of their high transmittance, tunable WF (2.14*–*5.65 eV), and metallic conductivity. Chen et al. introduced 2D Ti_3_C_2_ MXene nanosheets as an interlayer into all-inorganic CsPbBr_3_ PSCs for the first time [160]. The MXene interlayer was formed on the FTO/c-TiO_2_/CsPbBr_3_ substrate via spin-coating of the Ti_3_C_2_ MXene nanosheet dispersion solution (Fig. 13a–b). The selected area electron diffraction (SAED) pattern of Ti_3_C_2_ MXene nanosheets shows a few highly crystalline layers with the device structure of FTO/TiO_2_/CsPbBr_3_/Ti_3_C_2_ MXene/Carbon to achieve a better interfacial energy-level alignment, which aids in the elimination of the energy-level mismatch, speeds up hole extraction, and lowers recombination at the interface of the perovskite/carbon electrode. Additionally, the Ti_3_C_2_ MXene nanosheets’ surface functional groups offer powerful interactions between the MXene and under-coordinated Pb atoms. This significantly lessens the deep trap defects in the CsPbBr_3_ films. The device with the Ti_3_C_2_-MXene interlayer displays an exceptional initial PCE of ~ 9% (Fig. 13c), with a long-term stability of more than 1900 h in a moist environment and more than 600 h under heat circumstances [160].

### At the Interfaces Between Perovskites and ETLs

Proper device design and specialized interface engineering are required to improve optoelectronic characteristics and the charge extraction process at the selective electrodes to increase the PCE of PSCs. In 2019, Agresti et al. used 2D Ti_3_C_2_T_*x*_ MXene (T_*x*_ = –O, –OH, and –F) to tune the WF of the perovskite absorber and engineered a perovskite/ETL interface. It was observed that the OH-terminated surface of MXene reduced the WF of the perovskite, while the O-terminated surface increased the WF. This nonlinear behavior is reported in the literature [178]. Furthermore, light absorption was increased in MXene -doped perovskites. The addition of Ti_3_C_2_T_*x*_ to halide perovskite and TiO_2_ layers enables the adjustment of the materials' WFs without changing the other electronic characteristics. The PSC with MXene-doped perovskite active layer also showed higher PCE (17.4%) than that with the un-doped perovskite absorber layer (15.6%) [179]. Additionally, the band alignment between these layers can be altered using the dipole induced by Ti_3_C_2_T_*x*_ at the perovskite/ETL interface. WF tuning and interface engineering work together to significantly improve the PCE of MXene-modified PSCs to 20% with a *V*_*oc*_ of 1.09 V, a *J*_*sc*_ of 23.82 mA cm^−2^ and an FF of 77.6%, as well as a reduction in hysteresis compared to reference cells without MXene [179]. Chen et al. used Ti_3_C_2_T_*x*_ QDs (TQDs) to engineer a perovskite/TiO_2_ ETL interface and perovskite absorber and introduced Cu_1.8_S nanocrystals to optimize the Spiro-OMeTAD HTL in PSCs (Fig. 13d) [180]. The QDs with a diameter of about 5.2 nm and a thickness of ~ 1 nm in the absorber layer significantly contribute to the improved crystalline quality of the perovskite film, and large grain sizes are formed. Furthermore, TQDs improve electron extraction and collection at the perovskite/ETL. Cu_1.8_S improves the hole extraction at perovskite/HTL interfaces. With the improved *J*_*sc*_, *V*_*oc*_, and FF because of the synergistic effect of both TQD/Cu_1.8_S, the hysteresis-free PCE of PSCs significantly increased from 18.3 to 21.6% (*V*_*oc*_ of 1.14 V, *J*_*sc*_ of 24.12 mA cm^−2^, FF of 78.70%) (Fig. 13e). The TQD and/or Cu_1.8_S nanocrystals doping also significantly improves the long-term ambient and light stability of PSCs by improving perovskite crystallization, reducing HTL film aggregation and crystallization, and preventing ETL from ultraviolet-induced photocatalysis. The results show that TQD and Cu_1.8_S can function as ultrafast electron and hole tunnels for optoelectronic devices. The trap-filled limit voltage (*V*_*TFL*_) values of FTO/TiO_2_/Perovskite/PCBM/Ag (control), FTO/TiO_2_/MQD/Perovskite/PCBM/Ag (S1), and FTO/TiO_2_/MQD/MQD-Perovskite/PCBM/Ag (S2) electron-only devices were found to be 0.82, 0.66, and 0.59 V, respectively. The electron trap-state densities of perovskites are 1.59 × 10^16^ cm^−3^ (control), 1.28 × 10^16^ cm^−3^ (S1), and 1.15 × 10^16^ cm^−3^ (S2) (Fig. 13f). The large grain size and low grain boundary density in the perovskite film resulted in a lower trap density [180]. Crystal deformations, such as lattice strain at the surfaces and grain boundaries, owing to the soft perovskite lattice, affect the charge extraction-transfer dynamics and recombination, leading to a low PCE. Zhou et al. added an inorganic 2D Ti_3_C_2_Cl_*x*_ MXene to the bulk and surface of the CsPbBr_3_ film [90]. This method dramatically reduces the superficial lattice tensile strain. The expanded perovskite lattice is compressed and confined to act as lattice "tape", in which the Pb-Cl bond functions as "glue", and the 2D Ti_3_C_2_ immobilizes the lattice. This compression and confinement result from the strong interaction between Cl atoms in Ti_3_C_2_Cl_*x*_ and the under-coordinated Pb^2+^ in the CsPbBr_3_ lattice. Under light irradiation, the carrier transfer is affected because defects and strain are introduced to the perovskite film. Hence, a smaller number of holes is available at the perovskite top surface. The MXene interlayer, defective states, and grain boundaries are passivated, leading to the availability of charge carriers and thus enhanced photovoltage. The champion all-inorganic CsPbBr_3_ PSC is finally able to attain the PCE as high as 11.08% with an ultrahigh *V*_*oc*_ up to 1.702 V, which is the highest efficiency record for this type of PSCs to date. Additionally, the unencapsulated device exhibits almost unaltered performance at 85 °C for 30 days and at 80% RH for 100 days [90].

High-efficiency photovoltaic (PV) devices can be produced by adding 2D MXenes to the ETL of PSCs. However, the oxidation that results from the ambient fabrication of the ETLs causes an inevitable decline in the electrical characteristics of MXene. To enhance the photovoltaic performance of PSCs, Bati et al. used metallic single-walled carbon nanotubes (m-SWCNTs) to make MXene/SWCNT composites [181]. The champion device with a configuration of ITO/SnO_2_:MXene/SWCNT(2:1)/(FAPbI_3_)_*x*_(MAPbBr_3_)_1−*x*_/Spiro-OMeTAD/Au attained a maximum PCE of 21.42%, a *J*_*sc*_ of 25.09 mA cm^−2^, a *V*_*oc*_ of 1.073 V and an FF of 80% with the optimum composition. The improved PL and reduced charge transfer resistance confirmed a low trap density and improved charge extraction and transport characteristics owing to the improved conductivity facilitated by the presence of carbon nanotubes and decreased oxygen vacancies on the surface of SnO_2_. The MXene/SWCNTs approach offers a promising route to realize high-performance PSCs [125, 181].

**Fig. 14:**
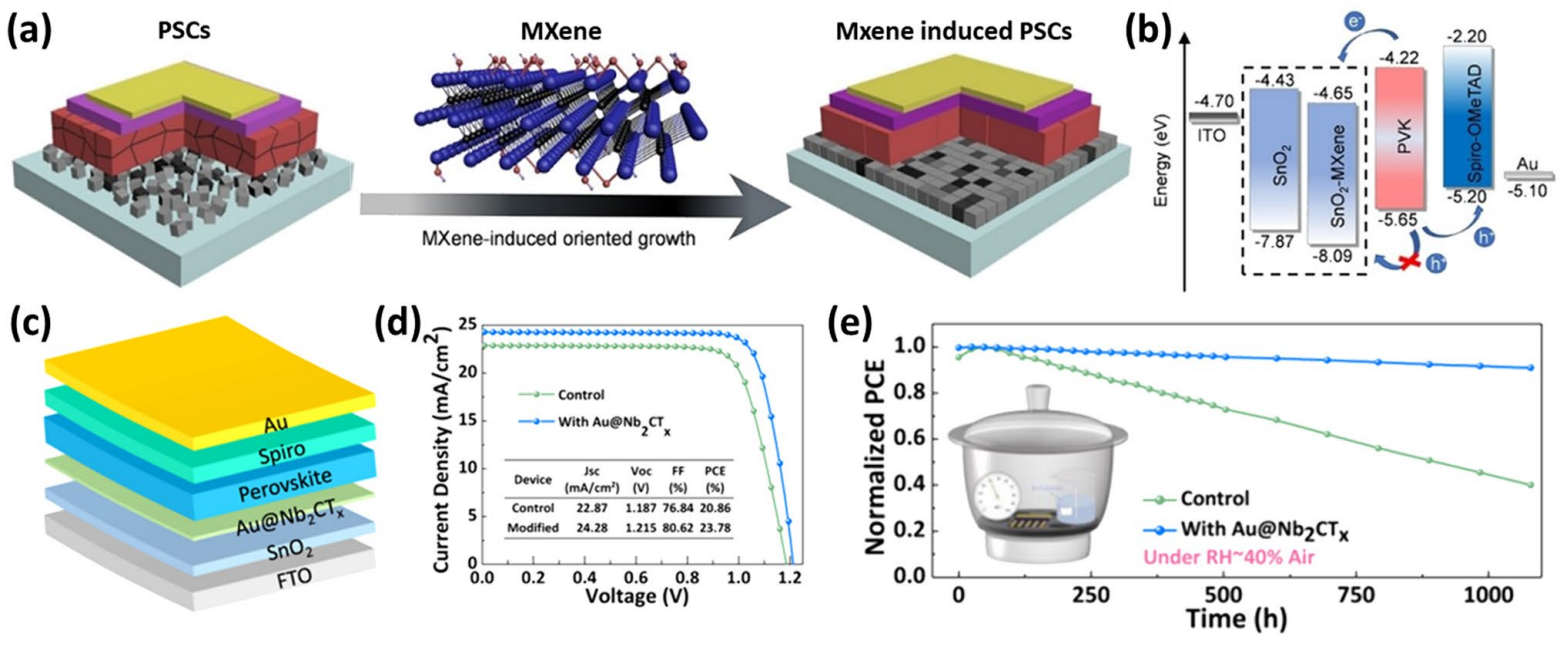
**a** Schematic of PSCs and MXene-PSCs films, MXene induces vertical growth of perovskite and improves the interfacial contact between perovskite and SnO_2_**. b** Energy level diagram of the device SnO_2_- MXene interface illustrating hindrance of hole transport [182]. Copyright © 2022 Wiley**. c** Structure of PSCs modified by Au@Nb_2_CT_*x*_- MXene**. d** *J* *V* curves of the control and Au@Nb_2_CT_*x*_-MXene-modified devices measured under the reverse scan**. e** Stability test of the control and Au@Nb_2_CT_*x*_- MXene-modified PSCs in ambient air under RH ∼ 40% for over 1000 h [184]. Copyright © 2023 American Chemical Society

As discussed earlier, the major causes of PSCs’ instability and low PCE are defects at the interfaces of perovskite thin films. Wu et al. used a composite ETL made of SnO_2_-MXene to enhance interfacial contact and passivate defects at the SnO_2_/perovskite interface in PSCs [182]. The newly developed MXene controls SnO_2_ dispersion and allows the perovskite film to grow vertically. The Lewis acid–base interaction of the -OH functional groups on the MXene surface and Sn atoms in SnO_2_ weakens the van der Waals interactions between neighboring crystals, impeding the accumulation of SnO_2_ nanocrystals. The lattice matching between MXene and perovskite nullifies the generated interfacial stress and induces vertical growth of perovskites (Fig. 14a). The average grain size of SnO_2_-MXene/perovskite (~ 1 μm) increased compared to the grain size of the control SnO_2_/perovskite (∼356 nm). Additionally, the conductivity of SnO_2_ increased from 1.85 × 10 to 9.62 × 10 S cm^*−*1^. This resulted in a higher current in MXene-PSCs (25.07 mA cm^*−*2^) than in pristine PSCs (24.16 mA cm^*−*2^). The lifetime of SnO_2_/perovskite (τ_1_ = 20.73 ns, τ_2_ = 80.24 ns) is longer than that of charge carriers in SnO_2_- MXene/perovskite (τ_1_ = 17.34 ns, τ_2_ = 48.20 ns). Hence charges are extracted faster in the SnO_2_-MXene ETL than in the pristine SnO_2_ ETL. Moreover, the MXene introduction also downshifts the valence band edge to −7.93 eV (SnO_2_-MXene) from −7.72 eV (SnO_2_), which indicates the suppression of hole recombination and hole migration to the ITO electrode (Fig. 14b). The PCE of the SnO_2_-MXene-based device with a structure of ITO/SnO_2−_ MXene/Perovskite/Spiro-OMeTAD/Au attained a PCE of 23.07%, with a *V*_*oc*_ of 1.13 eV, a *J*_*sc*_ of 25.07 mA cm^*−*2^, and an FF of 81.1%. The attained PCE is 15% higher than for the SnO_2_-based device (20.03%). Furthermore, even after 500 h of storage at 30*–*40% relative humidity in ambient air, the unencapsulated device still retained almost 90% of its initial PCE [182].

Another research group led by Liu et al. used Au@Nb_2_CT_*x*_-MXene in PSCs to nullify charge accumulation and interfacial defects at SnO_2,_ leading to poor charge transfer from the perovskite layer to the charge transport layer [183]. An Nb_2_CT_*x*_-MXene multilayer was obtained by Nb_2_AlC via selective etching of Al atoms by HF treatment, and a further MXene monolayer was derived from the multilayer via tetramethylammonium hydroxide (TMAOH) treatment. Further, Au nanoparticles were coated on MXenes to form Au@Nb_2_CT_x_-MXene (Fig. 14c). In PSCs, Nb_2_CT_*x*_- MXene increases conductivity (3.37 × 10^−6^ S cm^−1^), thus lowering Sn vacancies in the SnO_2_ layer's interstitial void and consequently lowering defect density and aligning the bandgap. With the inclusion of Au NPs, Au@Nb_2_CT_*x*_-MXene improves the perovskite film quality, controls the tensile tension of perovskites, and stifles Auger recombination. As a result, the Au@Nb_2_CT_*x*_-MXene-modified device exhibits a very high *V*_*oc*_ of 1.215 V, a *J*_*sc*_ of 24.28 mA cm^−2^, an FF of 80.62%, and a PCE of 23.78%, compared to the control device with Nb_2_CT_*x*_- MXene (PCE of 20.86%) (Fig. 14d). In addition, the unencapsulated devices preserve 90% of their initial PCE values after being exposed to air with a relative humidity of 40% for 1000 h and continue to function at or above 80% of their initial efficiency after being exposed to sunlight for 500 h (Fig. 14e) [183].

**Fig. 15:**
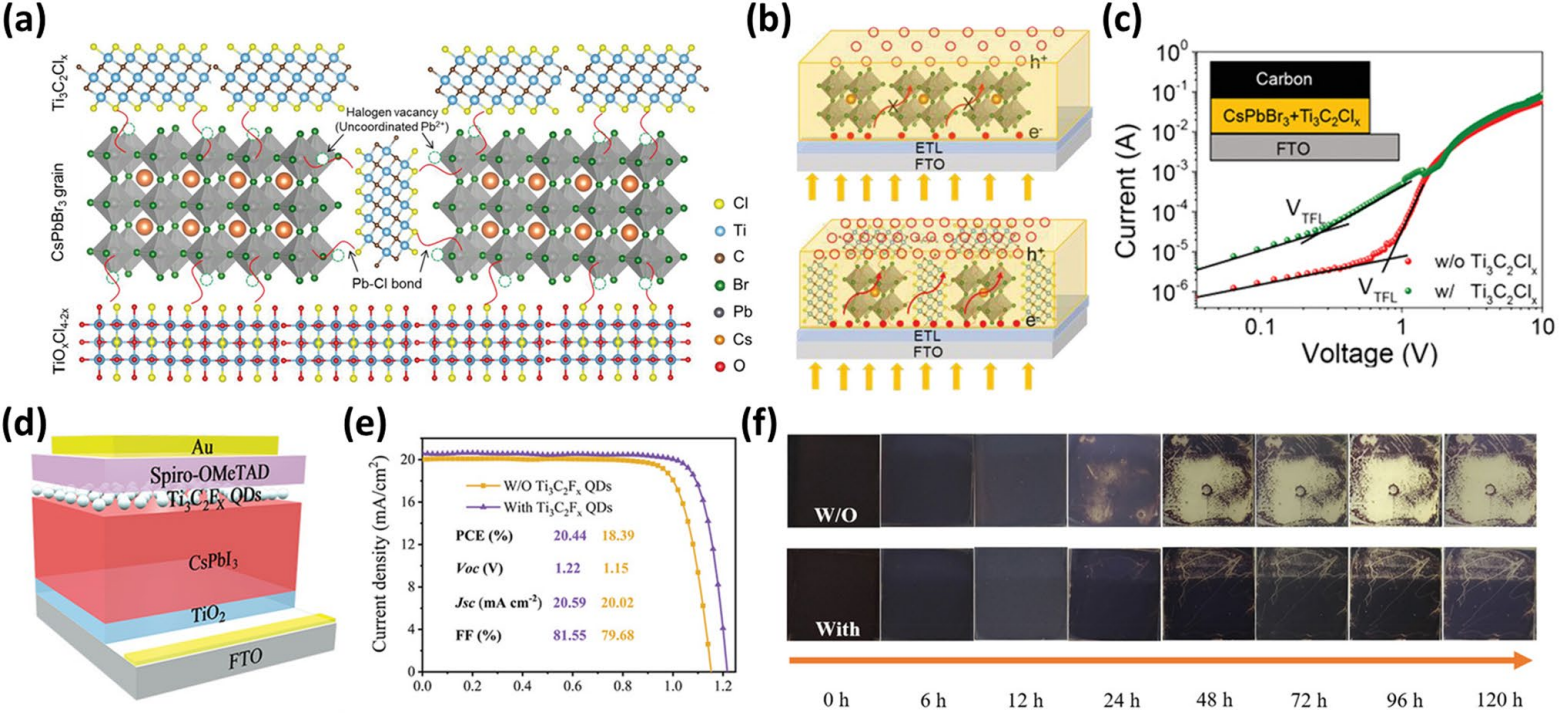
**a** Schematic diagram of full defect passivation in CsPbBr_3_ film by Ti_3_C_2_Cl_*x*_ MXene. **b** Illustration for the photogenerated carrier transfer in pristine and Ti_3_C_2_Cl_*x*_ containing perovskite films.** c** Dark *J–V* curves for the hole-only devices with and without Ti_3_C_2_Cl_*x*_ Mxene [90]. Copyright © 2021 Wiley–VCH GmbH.** d** Schematic image of a CsPbI_3_ PSC with the structure of FTO/TiO_2_/CsPbI_3_/Ti_3_C_2_F_*x*_/Spiro-OMeTAD/Au. **e** *J–V* curves of devices comprising pristine and Ti_3_C_2_F_*x*_ QDs treated CsPbI_3_ films. **f** Photographs of control and Ti_3_C_2_F_*x*_ QDs-treated CsPbI_3_ films aged in ambient air conditions (RH: ≈35%, T = 25 °C) [185]. Copyright © 2022 Wiley

As reported earlier by Zhou et al., 2D Ti_3_C_2_Cl_*x*_ MXene was inserted into the bulk and surface of CsPbBr_3_ film [90]. The 2D Ti_3_C_2_Cl_*x*_ MXene was deposited on the surface of SnO_2_ ETL. Inclusion of MXenes into bulk and surface of the device results in decreased defects and strains and leaving more holes for larger voltage (Fig. 15a–b). The defect reduction can be quantified in terms of the reduced trap-filled limit voltage (*V*_*TFL*_). The *V*_*TFL*_ decreases from 0.78 (pristine) to 0.56 V for Ti_3_C_2_Cl_*x*_ passivated perovskite film and the defect density (*n*_*t*_) decreased from 5.93 × 10^15^ to 1.92 × 10^15^ cm^−3^, respectively (Fig. 15c).

### At the Interfaces Between Perovskites and HTLs

CsPbI_3_ inorganic perovskites have garnered much interest for their outstanding thermal stability and suitable bandgap for tandem solar cells. However, due to nonradiative recombination, CsPbI_3_ PSCs show low PCEs and a high *V*_*oc*_ loss (0.5 *vs*. 0.3 V for hybrid perovskites) [184]. To improve the functionality of CsPbI_3_ PSCs, Xu et al. synthesized Ti_3_C_2_F_*x*_ MQDs with abundant Ti–F groups by a liquid-phase exfoliation technology and used them as interface passivators [185]. The Ti_3_C_2_F_*x*_ MQDs function as efficient passivators primarily in three ways: 1) p-type Ti_3_C_2_F_*x*_ MQDs can adjust the energy levels of perovskite films and provide a reliable pathway for hole transfer; 2) Ti_3_C_2_F_*x*_ MQDs can effectively passivate defects and reduce interfacial nonradiative recombination; and 3) Ti_3_C_2_F_*x*_ MQDs form a barrier layer. As a result, the champion CsPbI_3_ PSC treated with Ti_3_C_2_F_*x*_ MQDs (FTO/TiO_2_/CsPbI_3_/Ti_3_C_2_F_*x*_ MQDs/Spiro-OMeTAD/Au) displays excellent PCE of 20.4% (18.4% for the pristine) with high *V*_*oc*_ of 1.22 V, *J*_*sc*_ of 20.59 mA cm^−2^, and FF of 81.5% (Fig. 15d). The air stability of Ti_3_C_2_F_*x*_ MQDs treated CsPbI_3_ film is remarkably enhanced after 120 h of storage in air with an RH of 35%, as shown in the photographs of the films (Fig. 15e). In addition, after 600 h of storage in ambient air, the target device without encapsulation maintained 93% of its initial efficiency [185].

**Fig. 16:**
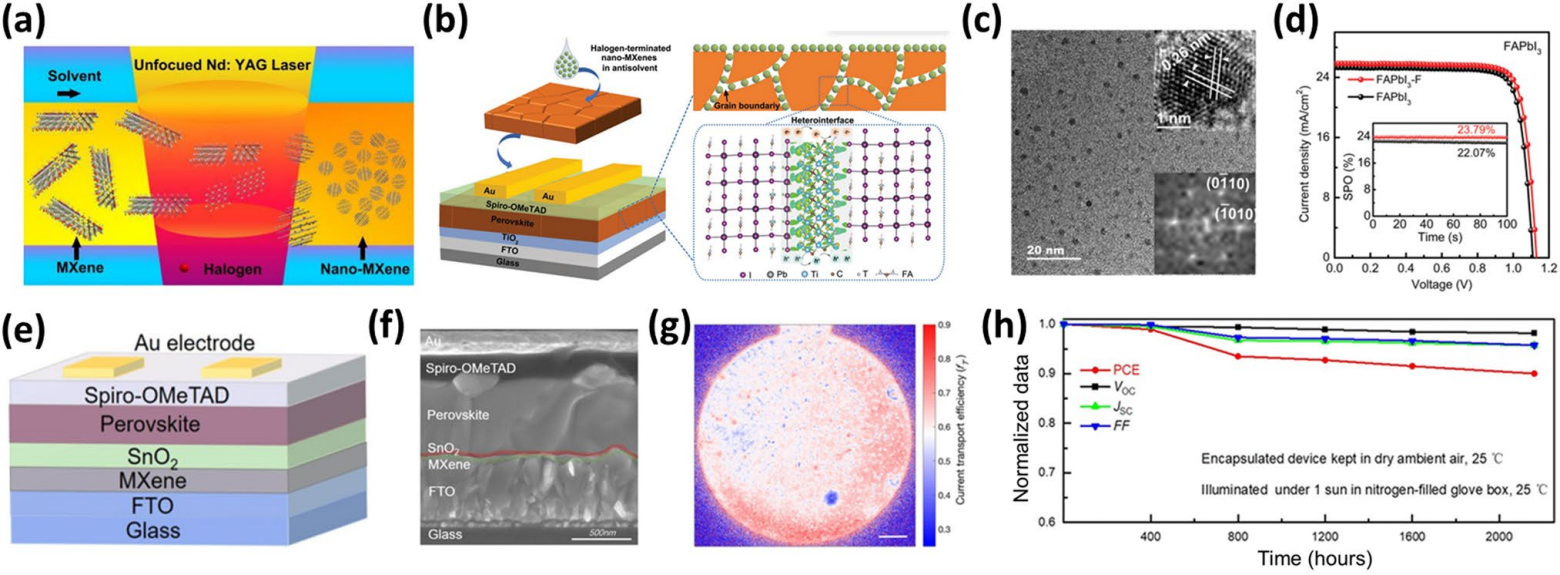
**a** Schematic diagram of nano-MXenes prepared via the pulsed laser irradiation. **b** Schematic of ionic-bonding heterointerface of hybrid perovskite via interfacial embedding of halogen-terminated nano-MXenes, Pb-T based ionic lattice anchoring and carrier dynamics modulation. **c** High-resolution transmission electron microscopy images and fast fourier transform patterns of nano-Ti_3_C_2_F_*x*_. **d** *J–V* curves of the champion PSCs based on FAPbI_3_ films and the stabilized power outputs of the corresponding devices (insets) [187]. Copyright © 2022 Wiley–VCH GmbH. **e–f** Device configuration and cross-sectional SEM image of the entire device. **g** Spatially resolved *f*_*T*_ maps of MXene-modified devices (scale bar, 1 mm). **h** Shelf lifetime tracking of the optimized PSC [191]. Copyright © 2020 American Chemical Society

Even at ambient temperatures, the perovskites based on formamidinium (FA) suffer an unnecessary spontaneous yellow phase transition yet promise a high PCE in photovoltaics. This has inspired significant efforts, which pose a formidable challenge to the soft perovskite lattice’s powerful anchoring. To effectively delay the lattice instability in FA-based perovskites, Guo et al. developed a rational design of interfacial ionic bonding between halogen (F, Cl, Br or I)-terminated nano-MXenes and perovskites [186]. They used pulsed laser irradiation to produce the halogen-terminated nano-MXenes using the green anti-solvent of ethyl acetate (Fig. 16a). The halognen-terminated nano-MXenes form heterointerfaces between nanocrystals and the perovskite (Fig. 16b–c). The strong heterointerface between perovskite and nano-MXenes also allows for efficient control of the WF of perovskite films, lowering of the interfacial charge transfer barrier, and effective modulation of deep-energy-level defects. The champion device with a structure of FTO/TiO_2_/FAPbI_3_/nano-Ti_3_C_2_T_*x*_/Spiro-OMeTAD/Au attained the highest PCE of 24.2%, a *J*_*sc*_ of 25.84 mA cm^−2^, a *V*_*oc*_ of 1.132 V and an FF of 82.6%. This is the highest efficiency utilizing MXene in PSCs reported so far. The stabilized power outputs for the pristine and the modified PSCs are 22.07% and 23.79%, respectively (Fig. 16d). These advantages enable unencapsulated FA-based perovskite solar cells to maintain over 90% of their initial efficiency after operation at maximum power point under continuous illumination for 1000 h and more than 85% of their initial efficiency even after annealing for 1000 h at 85 °C in an inert atmosphere [186].

### At the Interfaces Between ETLs and Electrodes

The high annealing temperature (> 450 °C), critical photo-instability of TiO_2_, and spontaneous aggregation of SnO_2_ hinder the scale-up of the commercial application of PSCs [187–189]. Furthermore, inadequate charge extraction and hysteresis are due to the energy-level mismatch between the conduction band minimum (CBM) of SnO_2_ and the WF of FTO at the interface. Hence interface engineering is essential for charge extraction in PSCs. Wang et al. introduced a strong interface interacting with 2D carbide MXene to improve the electron mobility and charge transfer ability of SnO_2_ ETL (Fig. 16e–f) [190]. The MXene-modified SnO_2_ ETL induced a preferable growth orientation route for perovskite films. It reduced the trap density and thereby reduced the non-radiative recombination. As shown in Fig. 16g, the MXene-modified device has a few localized defects, and the carrier transport is homogeneously enhanced, as reflected by the current transport efficiency (*f*_*T*_*)* values over the active area of the entire device. Furthermore, the modified SnO_2_/perovskite interface provided a better platform for charge extraction and transfer. The PSC with MXene-modified SnO_2_ and a device structure of FTO/(0.5 mg mL^−1^ MXene)/SnO_2_/Perovskite/Spiro-OMeTAD/Au achieved a PCE of 20.7% with ultralow saturated current density and negligible hysteresis. Moreover, the champion PSC demonstrated an excellent shelf lifetime by retaining 90% of its initial PCE after 3 months. However, no significant degradation is observed in the *V*_*oc*_ of the PSC with an MXene interlayer (Fig. 16h) [190].

The electrical conductivity of 2D MXene can be adjusted by changing the termination groups and applying controlled oxidation, which has enormous promise for solar applications. Yang et al. added in-situ oxidized Ti_3_C_2_T_*x*_ (O-Ti_3_C_2_T_*x*_) MXene to create a nanoscale heterojunction with SnO_2_ that was used as an ETL in a MAPbI_3_ PSC with a device structure of FTO/O-Ti_3_C_2_T_*x*_/SnO_2_/MAPbI_3_/Spiro-OMeTAD/Ag [191]. The properties of Ti_3_C_2_T_*x*_ are modified to have their energy levels well-matched with the perovskite by taking advantage of the change from metallicity to semiconductivity during the oxidation process. Additionally, the O-Ti_3_C_2_T_*x*_ insertion increased the SnO_2_ ETL electron mobility. As a result, the O-Ti_3_C_2_T_*x*_/SnO_2_ heterojunction provided better electron extraction and decreased recombination between the ETL and the perovskite, increased PCE from 17.7 to 20.1%, and enhanced stability with preserving 80% of its initial PCE in air [191].

## Prospects and Conclusions

**Fig. 17:**
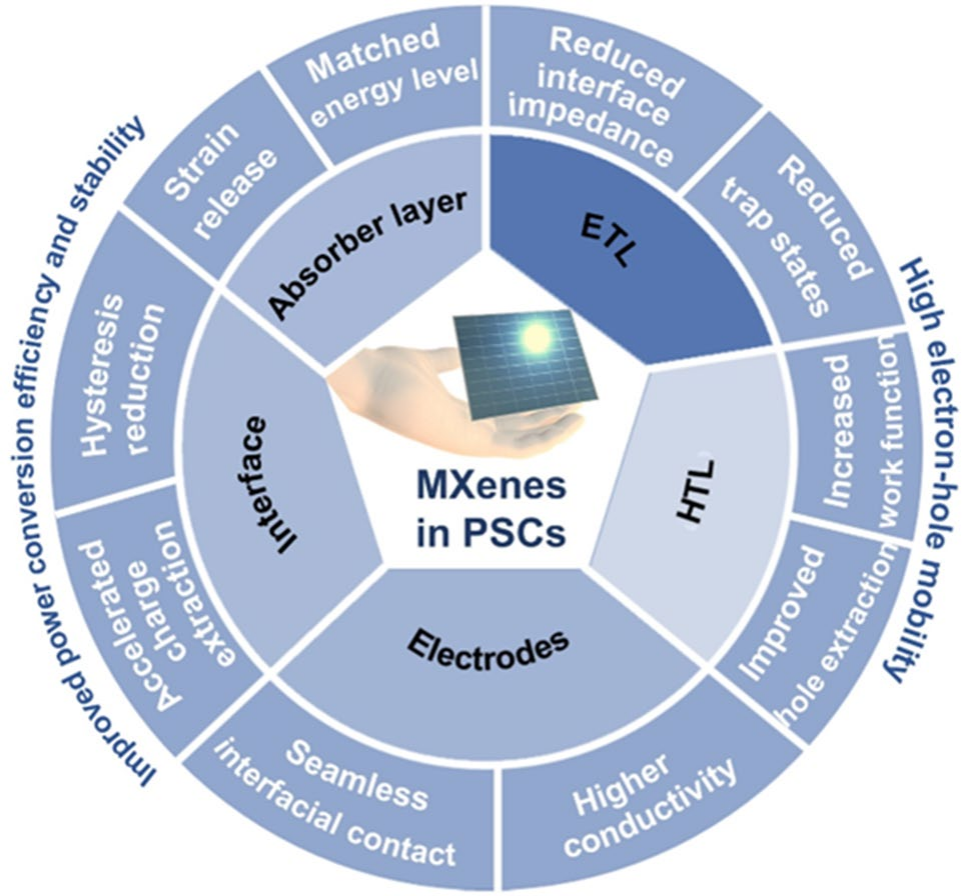
Positions and functions of MXenes in PSCs

MXenes have significantly impacted the device performance and stability of the PSCs. The roles and functions of MXenes in PSCs are summarized as shown in Fig. 17**.** As an application, a detailed overview of MXenes in PSCs starting from the first report in 2018 to recent advances, has been provided. MXenes play multiple roles as different components of PSC. The roles are broadly divided into five categories depending on their functions: additive in perovskite absorber layers, ETLs/additives in ETLs, HTLs/additives in HTL, electrodes, and interfacial layers. As additives in absorbers, MXenes retard the crystallization rate and enlarge the crystal sizes of perovskites by controlling the morphology. Furthermore, MXenes mitigate perovskite lattice instability and passivate the bulk and surface defects, prolonging the carrier lifetimes and reducing charge recombination. In addition, these materials induce hydrophobic features on the surface of the absorber layer and prevent deprotonation of protonated organic amine in perovskites. Most importantly, MXenes can tune the work function of the perovskite absorber layer for efficient charge transfer. The combined effect of these features improves the PCE and stability of the PSCs. Similarly, as/in ETL, MXenes tune the work functions of the ETLs for well-matched band alignment with the perovskite layer to facilitate smooth electron transport. These observations are further supported by the improved electron mobilities and the reduced interface recombination for the ETL/perovskite. In addition, MXenes mitigate the defects in the ETL layer, which is a prerequisite for improved crystallinity of the next perovskite layer. As electrodes, MXenes pose as emerging materials in transparent hybrid electrodes in PSCs. MXenes as/or in HTLs provide a superior hole transfer path, improve hole extraction, and decrease charge transfer resistance at the HTL/perovskite interface via tuning the WF of the HTLs. The presence of MXenes in electrodes provides improved adhesion to the substrates and superior continuous conductive paths for improved electrical conductivity. In addition, because of their low square resistance, MXenes act as promising electrodes. MXenes are also used as additional interfacial layers in ETL/electrodes, perovskite/electrode, perovskite/ETL, and perovskite/HTL layers. These layers help in efficient charge extractions and transfers via improved interfacial energy alignment, reduce recombination (trap defects) at the interfaces, provide a well-controlled growth platform for smooth perovskite films, and passivate perovskite grains for improved film quality. These essential features provide a promising route for highly efficient and stable PSCs. Different roles of MXenes and their photovoltaic performance in PSCs are summarized in Tables 1, 2, 3, 4, and 5. Until now, MXene-assisted PSCs have attained the highest PCE of 24.17% and the highest reported stability time reported so far is 2000 h. In addition, the PSCs with a large area of 0.99 cm^2^ and the minimodules with the size of 25 cm^2^ are reported so far. However, MXene’s applications in perovskite solar cells are in their infancy, and many advancements are yet to come. Several distinct MXenes with varying compositions, surface terminations, band topologies, and WFs have been reported. However, only a limited number of MXenes such as Ti_3_C_2_T_*x*_ (T_*x*_ = –O, –OH, –F, –Cl, –Br, –I, –NH_2_), V_2_CT_*x*_, Mo_2_C, and Nb_2_CT_*x*_ have been reported in PSCs so far. Hence theoretical studies are required to further understand the charge transport/extraction behavior and WF modification of new MXene compositions, MXene/perovskite, MXene/ETL, and MXene/HTL interfaces. A more detailed theoretical investigation is required to elucidate the optical, electrical, and mechanical properties of MXenes, effects of various termination groups of MXenes, surface modifications, variation of WF, and energy-level alignment on the photovoltaic parameters of MXene-based PSCs. In addition, different new synthesis routes and post-treatments such as laser irradiation, UV-ozone treatment, etc. need to be explored to obtain high quality pure and functionalized MXenes. MXenes should be transparent in the photovoltaic response range of PSCs in addition to having strong flexibility and high electrical conductivity. In addition, the benefits that MXenes provide in terms of material characteristics and performance should ensure affordable price, environmental compatibility, long-term stability, and sustainable manufacturing in upscaling the production of MXene and its commercial uses in PSCs (small size and large size) and solar cell modules [192]. Furthermore, the additional cost due to MXenes inclusion in PSCs needs to be evaluated. As a next step, a plethora of unexplored MXenes materials needs to be studied and optoelectronic properties are to be optimized to improve both PCE and stability of PSCs further. Apart from PSCs, the MXenes have drawn interest in other perovskite-based optoelectronic devices such as photodetectors, photodiodes, phototransistors, and plasmonic, etc. [193]. MXenes are used in photodetectors (PDs) in various roles, such as transparent electrodes, Schottky contacts, light absorbers, and plasmonic materials [194]. Ti_3_C_2_T_*x*_ quantum dots (TQDs) are used to boost the charge carriers of 2D perovskite Ca_2_Nb_3_O_10_ (CNO) nanosheets (NS) and enhance the performance of the TQD-modified 2D perovskite CNO NS PD. Other such examples are CsPbBr_3_ NSs/Ti_3_C_2_T_*x*_ MXene and CsPbBr_3_ NCs/Ti_3_C_2_T_*x*_ MXenes nanocomposite PDs [195]. Similarly, plasmonic Nb_2_CTx MXene-MAPbI_3_ heterostructure is reported for self-powered visible-NIR photodiodes [196].

**Table 1:** Role of MXenes as additives in the perovskite absorber layers in PSCs [The letter “C” in the bracket stands for the control (reference or pristine) device)]

Device structure	*V*_*oc*_ [V]	*J*_*sc*_ [mA cm^−2^]	FF [%]	PCE [%]	Refs.
ITO/SnO_2_/MAPbI_3_/Spiro-OMeTAD/Au (C)	1.00	20.67	75	15.58	[31]
ITO/SnO_2_/MAPbI_3_:Ti_3_C_2_T_*x*_/Spiro-OMeTAD/Au	1.03	22.26	76	17.41	
ITO/PTAA/CsPbI_3_/CPTA/BCP/Ag (C)	1.18	19.05	80.33	18.05	[88]
ITO/PTAA/CsPbI_3_/O-Ti_3_C_2_T_*x*_-CsPbI_3_/CPTA/ BCP/Ag	1.21	19.85	81.61	19.69	
FTO/SnO_2_-TiO_*x*_Cl_4−2*x*_/CsPbBr_3_:Ti_3_C_2_Cl_*x*_/Ti_3_C_2_Cl_*x*_/Carbon (C)	1.59	7.32	79.9	9.18	[90]
FTO/SnO_2_-TiO_*x*_Cl_4−2*x*_/CsPbBr_3_:Ti_3_C_2_Cl_*x*_/ Ti_3_C_2_Cl_*x*_/Carbon	1.702	7.87	82.7	11.08	
ITO/NiO/MAPbI_3_/PCBM/BCP/Ag (C)	1.09	21.41	77	17.97	[91]
ITO/NiO/MAPbI_3_ + Ti_3_C_2_T_*x*_/PCBM/BCP/Ag	1.08	22.33	77	18.57	
ITO/SnO_2_/(BA)_2_(MA)_4_Pb_5_I_16_/Spiro-OMeTAD/Ag (C)	1.09	18.84	66.70	13.69	[96]
ITO/SnO_2_/(BA)_2_(MA)_4_Pb_5_I_16_-Ti_3_C_2_T_*x*_ /Spiro-OMeTAD/Ag	1.11	20.87	67.84	15.71	
ITO/SnO_2_/FA_1-*x*_MA_*x*_PbI_3-*y*_Br_*y*_/Spiro-OMeTAD/Au (C)	1.07	25.02	71	19.03	[97]
ITO/SnO_2_/FA_1-*x*_MA_*x*_PbI_3-*y*_Br_*y*_:Cs-Ti_3_C_2_T_*x*_/Spiro-OMeTAD/Au	1.10	26	76	21.57	
FTO/SnO_2_/Rb_0.05_Cs_0.05_(FA_0.83_MA_0.17_)_0.90_Pb(I_0.83_Br_0.17_)_3_/Spiro-OMeTAD/Au (C)	1.14	21.16	77.85	18.78	[98]
FTO/SnO_2_/Rb_0.05_Cs_0.05_(FA_0.83_MA_0.17_)_0.90_Pb(I_0.83_Br_0.17_)_3_:Ti_3_C_2_T_*x*_ QDs/Spiro-OMeTAD/Au	1.19	22.27	80.42	21.31	
FTO/SnO_2_/MAPbI_3_/Spiro-MeOTAD/Au (C)	1.08	21.53	70.47	16.45	[106]
FTO/SnO_2_/MAPbI_3_:Ti_3_C_2_T_*x*_/Spiro-MeOTAD/Au	1.12	23.48	73.66	19.27	
FTO/c-TiO_2_/m-TiO_2_/D-149-Cs_2_AgBiBr_6_/Spiro-OMeTAD/Ag (C)	0.716	8.20	69.1	4.06	[107]
FTO/c-TiO_2_/m-TiO_2_/D149-Cs_2_AgBiBr_6_@Ti_3_C_2_T_*x*_/Spiro-OMeTAD/Ag	0.722	8.85	70.1	4.47	
FTO/c-TiO_2_/m-TiO_2_/m-ZrO_2_/MAPbI_3_/Carbon (C)	0.80	23.03	61.81	11.35	[108]
FTO/c-TiO_2_/m-TiO_2_/m-ZrO_2_/MAPbI_3_:Ti_3_C_2_T_*x*_/Carbon	0.81	17.64	61.12	13.62	
ITO/PTAA/MAPbI_3_/PCBM:BCP/Ag (C)	1.00	20.60	73.20	15.01	[110]
ITO/PTAA/MAPbI_3_:V_2_CT_*x*_/PCBM:BCP/Ag	1.03	23.12	73.90	17.61	
ITO/P-Nb_2_CT_*x*_/FA_0.85_Cs_0.15_PbI_3_/Spiro-OMeTAD/Ag (C)	1.054	23.82	71.49	17.95	[112]
ITO/T-Nb_2_CT_*x*_/FA_0.85_Cs_0.15_PbI_3_:T-Nb_2_CT_*x*_/Spiro-OMeTAD/Ag	1.124	25.07	77.36	21.79	
FTO/c-TiO_2_/m-TiO_2_/CsFAMAIBr perovskite/Spiro-OMeTAD/Au (C)	1.10	23.11	71.83	18.3	[180]
FTO/c-TiO_2_/m-TiO_2_/Ti_3_C_2_T_*x*_ QD/Ti_3_C_2_T_*x*_ QD + CsFAMAIBr perovskite/Spiro-OMeTAD/Au	1.12	23.51	74.62	20.72

**Table 2:** Role of MXenes as ETL/ additives in ETL in PSCs [The letter “C” in the bracket stands for the control (reference or pristine) device)]

Device structure	*V*_*oc*_ [V]	*J*_*sc*_ [mA cm^−2^]	FF [%]	PCE [%]	Refs.
ITO/NiO/MAPbI_3_/PCBM/BCP/Ag (C)	1.09	21.41	77	17.97	[91]
ITO/NiO/Ti_3_C_2_T_*x*_ doped- MAPbI_3_/MXene-PCBM/BCP/Ag	1.09	22.88	77	19.20	
FTO/SnO_2_/(FAPbI_3_)_0.97_(MAPbBr_3_)_0.03_/Spiro-OMeTAD (C)	1.07	21.88	71.90	16.83	[120]
FTO/MDCN air & N_2_/(FAPbI_3_)_0.97_(MAPbBr_3_)_0.03_/Spiro-OMeTAD	1.10	24.16	74.05	19.14	
ITO/SnO_2_/MA_0.15_FA_0.85_PbI_*x*_Br_(3−*x*)_/Spiro-OMeTAD/Ag (C)	1.111	20.65	70.99	16.28	[121]
ITO/SnO_2_/Ti_3_C_2_T_*x*_/MA_0.15_FA_0.85_PbI_*x*_Br_(3−*x*)_/Spiro-OMeTAD/Ag	1.113	23.65	76.01	20.35	
FTO/TiO_2_/Cs_2_AgBiBr_6_/Spiro-OMeTAD /MoO_3_/Ag (C)	0.93	3.29	65	2.00	[122]
FTO/Ti_3_C_2_T_*x*_@TiO_2_ (0.2 wt%)/Cs_2_AgBiBr_6_/Spiro-OMeTAD /MoO_3_/Ag	0.96	4.14	70	2.81	
ITO/NiO_*x*_/MAPbI_3_/PC_61_BM/BCP/Ag (C)*	1.04	21.53	72	15.55	[123]
ITO/NiO_*x*_/MAPbI_3_/Ti_3_C_2_T_*x*_-PC_61_BM/ BCP/Ag*	1.06	22.90	77	18.37	
ITO/NiO_*x*_/MAPbI_3_/Ti_3_C_2_T_*x*_/BCP/Ag*	0.72	5.47	50	2.01	
FTO/SnO_2_/MAPbI_3_/Spiro-OMeTAD/Ag (C)	1.06	23.04	72.88	17.78	[124]
FTO/SnO_2_-Ti_3_C_2_ MXene/MAPbI_3_/Spiro-OMeTAD/Ag	1.07	23.44	75.86	18.84	
ITO/MAPbI_3_/Spiro-OMeTAD/Ag (C)	0.80	15.87	40	5	[111]
ITO/Ti_3_C_2_T_*x*_/MAPbI_3_/Spiro-OMeTAD/Ag	1.08	22.63	70	17.17	
ITO/SnO_2_/CsFAMAIBr perovskite/Spiro-OMeTAD/Ag (C)	1.111	24.71	69.1	18.96	[125]
ITO/SnO_2_-Nb_2_C/CsFAMAIBr perovskite/Spiro-OMeTAD/Ag	1.138	25.29	79.5	22.86	
ITO/P-Nb_2_CT_*x*_/FA_0.85_Cs0_.15_PbI_3_/Spiro-OMeTAD/Ag (C)	1.054	23.82	71.49	17.95	[112]
ITO/T-Nb_2_CT_*x*_/FA_0.85_Cs_0.15_PbI_3_/Spiro-OMeTAD/Ag	1.117	24.55	73.79	20.23	
TO/T-Nb_2_CT_*x*_/FA_0.85_Cs_0.15_PbI_3_ + T-Nb_2_CT_*x*_/Spiro-OMeTAD/Ag	1.124	25.07	77.36	21.79	
FTO/TiO_2_:SnO_2_:Ti_3_C_2_T_*x*_/(FAPbI_3_)_0.97_(MAPbBr_3_)_0.03_/Spiro-OMeTAD/Au	1.10	22.03	77.78	18.9	[113]
ITO/SnO_2_/FAPbI_3_/Spiro-OMeTAD/MoO_*x*_/Ag (C)	1.093	25.22	81.11	22.36	[126]
ITO/Ti_3_C_2_T_*x*_-H-doped SnO_2_/FAPbI_3_/Spiro-OMeTAD/MoO_x_/Ag	1.121	25.49	84.42	24.12	
ITO/SnO_2_/FA_0.9_MA_0.05_Cs_0.05_PbI_0.98_Br_0.02_/Spiro-MeOTAD/MoO_3_/Au (C)	1.140	24.26	75.8	20.96	[127]
ITO/SnO_2_-Ti_3_C_2_T_*x*_ QDs/FA_0.9_MA_0.05_Cs_0.05_PbI_0.98_Br_0.02_/Spiro-MeOTAD /MoO_3_/Au	1.172	24.96	79.8	23.34	
ITO/MAPbI_3_/Spiro-OMeTAD/Ag (C)	1.03	20.61	70	14.86	[114]
ITO/HO-Ti_3_C_2_T_*x*_@Ti_3_C_2_T_*x*_/MAPbI_3_/Spiro-OMeTAD/Ag	1.07	23.11	74	18.29	
FTO/c-TiO_2_: Ti_3_C_2_T_*x*_/m-TiO_2_:Ti_3_C_2_T_*x*_/Ti_3_C_2_T_*x*_/MAPbI_3_:Ti_3_C_2_T_*x*_/Spiro-OMeTAD/Au	1.09	23.82	77.6	20.14	[179]
FTO/c-TiO_2_/m-TiO_2_/Ti_3_C_2_T_*x*_ QD/CsFAMAIBr perovskite/Spiro-OMeTAD/Au	1.12	23.51	74.62	19.65	[180]

The sign"*” represents the maximum values of the photovoltaic parameters

**Table 3:** Role of MXenes as HTL/ additives in HTL in PSCs [The letter “C” in the bracket stands for the control (reference or pristine) device)]

Device structure	*V*_*oc*_ [V]	*J*_*sc*_ [mA cm^−2^]	FF [%]	PCE [%]	Refs.
ITO/MAPbI_3_/PCBM/Ag (C)	1.047	20.16	73.57	15.53	[134]
ITO/Nb_2_CT_*x*_/MAPbI_3_/PCBM/Ag	1.128	23.06	79.75	20.74	
ITO/ Ti_3_C_2_T_*x*_-SCA/MAPbI_3_/PC_61_BM/Ag	0.87	21.30	73.47	13.65	[139]
ITO/PEDOT:PSS/MAPbI_3_/PCBM/LiF/Al (C)	0.884	18.145	59	9.28	[153]
ITO/Mo_2_C-CNT@PEDOT:PSS/MAPbI_3_/PCBM/LiF/Al*	0.881	22.219	62	12.14	
ITO/PEDOT:PSS/MAPbI_3_/PCBM/LiF/Al (C)	0.904	18.59	56.73	9.31	[154]
ITO/Ti_3_C_2_T_*x*_/WO_3_@PEDOT:PSS/MAPbI_3_/PCBM/LiF/Al*	0.924	22.73	60.71	12.38	

The sign"*” represents the maximum values of the photovoltaic parameters

**Table 4:** Role of MXenes as electrodes in PSCs [The letter “C” in the bracket stands for the control (reference or pristine) device)]

Device structure	*V*_*oc*_ [V]	*J*_*sc*_ [mA cm^−2^]	FF [%]	PCE [%]	Refs.
FTO/TiO_2_/MAPbI_3_/Coal (C)	0.84	21.39	60	10.87	[157]
FTO/TiO_2_/MAPbI_3_/Ti_3_C_2_T_*x*_	0.95	22.96	63	13.83	
FTO/c-TiO_2_/CsPbBr_3_/Carbon + CNTs (C)	1.250	5.81	65.68	4.77	[161]
FTO/c-TiO_2_/CsPbBr_3_/Carbon + CNTs + Ti_3_C_2_−MXene	1.357	7.16	72.97	7.09	
PET/AgNW:Ti_3_C_2_T_*x*_/NiO_*x*_/Cs_0.05_FA_0.85_MA_0.10_Pb(I_0.97_Br_0.03_)_3_/PC_61_BM/Ag	1.06	25.16	75.52	20.22	[176]

**Table 5:** Role of MXenes as interfacial layers in PSCs [the letter “C” in the bracket stands for the control (reference or pristine) device]

Device structure	*V*_*oc*_ [V]	*J*_*sc*_ [mA cm^−2^]	FF [%]	PCE [%]	Refs.
FTO/SnO_2_-TiO_*x*_Cl_4−2*x*_/CsPbBr_3_:Ti_3_C_2_Cl_*x*_/Ti_3_C_2_Cl_*x*_/Carbon	1.702	7.87	82.7	11.08	[90]
ITO/NiO_*x*_/MAPbI_3_/Ti_3_C_2_T_*x*_/PC_61_BM/BCP/Ag*	1.05	20.74	74	15.99	[123]
FTO/TiO_2_/CsPbBr_3_/Carbon (C)	1.387	7.10	72.18	7.11	[160]
FTO/TiO_2_/CsPbBr_3_/Ti_3_C_2_ MXene/Carbon	1.444	8.54	73.08	9.01	
FTO/c-TiO_2_:Ti_3_C_2_T_*x*_/m-TiO_2_:Ti_3_C_2_T_*x*_/Ti_3_C_2_T_*x*_/MAPbI_3_:Ti_3_C_2_T_*x*_/Spiro-OMeTAD/Au	1.09	23.82	77.6	20.14	[179]
ITO/SnO_2_/FA_*x*_MA_1-*x*_I_3-*y*_Br_*y*_/Spiro-OMeTAD/Au (C)	1.043	24.71	73	18.84	[181]
ITO/SnO_2_/Ti_3_C_2_T_*x*_:m-SWCNT/FA_*x*_MA_1-*x*_I_*y*_Br_3-*y*_/Spiro-OMeTAD/Au	1.073	25.09	80	21.42	
ITO/SnO_2_/CsFAMAIBr perovskite/Spiro-OMeTAD/Au (C)	1.09	24.16	75.18	20.03	[182]
ITO/SnO_2_-MXene/CsFAMAIBr perovskite/Spiro-OMeTAD/Au	1.13	25.07	81.1	23.07	
FTO/SnO_2_/Nb_2_CT_*x*_/CsFAMAIBr perovskite/Spiro-OMeTAD/Au	1.187	22.87	76.84	20.86	[183]
FTO/SnO_2_/Au@Nb_2_CT_*x*_/CsFAMAIBr perovskite/Spiro-OMeTAD/Au	1.215	24.28	80.62	23.78	
FTO/SnO_2_/CsPbI_3_/Spiro-OMeTAD/Au (C)	1.15	20.02	79.68	18.39	[185]
FTO/SnO_2_/CsPbI_3_/Flourine-functionalized Ti_3_C_2_T_*x*_ QDs/Spiro-OMeTAD/Au (C)	1.22	20.59	81.55	20.44	
FTO/TiO_2_/FAPbI_3_/Spiro-OMeTAD/Au (C)	1.102	25.30	79.81	22.25	[186]
FTO/TiO_2_/FAPbI_3_/nano-Ti_3_C_2_F_*x*_/Spiro-OMeTAD/Au	1.132	25.84	82.63	24.17	
FTO/Ti_3_C_2_T_*x*_/SnO_2_/ Cs_0.05_FA_0.76_MA_0.19_PbI_2.715_Br_0.285_/Spiro-OMeTAD/Au	1.11	24.34	76.4	20.65	[190]
FTO/SnO_2_/MAPbI_3_/Spiro-OMeTAD/Ag (C)	1.046	23.22	72.8	17.68	[191]
FTO/O-Ti_3_C_2_T_*x*_/SnO_2_/MAPbI_3_/Spiro-OMeTAD/Ag	1.075	23.88	74.8	20.09	

The sign"*” represents the maximum values of the photovoltaic parameters

In recent years, perovskite solar cells have undergone significant scientific developments. Low-cost, low-temperature solution-based fabrication processes, advances in multi-cation and -anion perovskites, improvements in all-inorganic perovskites, Pb-free perovskites, application of 2D/3D perovskites, defects passivation via solvent engineering, compositional engineering, interface engineering, device engineering, consistent improvements in the photovoltaic performance (PCE and stability) of tandem solar cells, solar concentrators incorporating scattering effects and spectral modification, and flexibility of perovskite solar cells hold the promise for scalability and large-scale commercialization of PSCs with high performance-to-cost ratios. In this review article, we have succinctly analyzed the incorporation of 2D MXene materials into the perovskite absorber layer, ETL/HTL, electrode, and interfaces. High optical transparency, wide tunable work function via functionalization of surface terminal groups, flexibility and superior mechanical properties of MXenes and their optimum concentrations are the key factors in producing high quality and uniform films, reducing trap states density and improving charge carrier extraction and collection capabilities in PSCs.These features present MXenes as tremendous potential candidates for PSC applications. This review highlights the recent advances of MXenes in PSCs that is simply a fractional part, and many key improvements in the MXene-modified PSCs are about to come in the near future.
